# Hierarchically Architected 3D-Printed Hydrogel Evaporators Enable Synergistic Salt Management and Photocatalytic Purification

**DOI:** 10.1007/s40820-026-02206-8

**Published:** 2026-05-11

**Authors:** Xin Yang, Xinqi Guo, Yankuan Tian, Rong Zhou, Yifei Gong, Chengming Zhang, Feng Ji, Liu Liu, Faxue Li, Ruiyun Zhang, Jianyong Yu, Tingting Gao

**Affiliations:** 1 Key Laboratory of Textile Science & Technology, Ministry of Education, College of Textiles, Donghua University, Shanghai, 201620 People’s Republic of China; 2 Innovation Center for Textile Science and Technology, Donghua University, Shanghai, 201620 People’s Republic of China; 3 Key Laboratory of Multifunctional Nanomaterials and Smart Systems, Suzhou Institute of Nano-Tech and Nano-Bionics, Chinese Academy of Sciences, Suzhou, 215123 People’s Republic of China; 4 College of Textiles and Fashion, Hunan Institute of Engineering, Xiangtan, 411104 People’s Republic of China

**Keywords:** Hydrogel evaporators, 3D printing, Salt resistance, Metal–organic framework, Solar desalination

## Abstract

Interfacial solar-driven water evaporation offers a sustainable route to clean water production, but faces critical challenges of salt accumulation and organic contamination in complex industrial wastewater treatment. To address these challenges, we engineered a multifunctional 3D-printed hydrogel evaporator with vertically aligned grid architectures and hierarchical porosity. This unique structure promotes rapid water replenishment and Marangoni-driven salt back-diffusion through millimeter-scale channels, effectively preventing salt crystallization. By integrating carbon black and the metal–organic framework PCN-224 into the printing ink, we constructed a dual-functional photothermal–photocatalytic system. This synergistic combination not only enhances light absorption and photothermal conversion but also significantly reduces the water evaporation enthalpy. Coupled with heat-accelerated reaction kinetics, the system achieves efficient broad-spectrum photocatalytic degradation of organic pollutants. The resultant composite evaporator attains a high water evaporation rate of 2.04 kg m⁻^2^ h⁻^1^ under one-sun illumination, maintaining stable performance across a wide salinity range. Simultaneously, it degraded 96.5% of rhodamine B within 60 min under 1.5 kW m⁻^2^ irradiation. This work demonstrates a synergistic strategy for simultaneous solar water production and purification, providing an efficient and environmentally friendly solution for advanced wastewater treatment.

**Supplementary Information:**

The online version contains supplementary material available at 10.1007/s40820-026-02206-8.

## Highlights

- 3D-printed low-tortuosity hydrogel enables efficient vapor and salt transport.
- Photothermal–photocatalytic synergy boosts evaporation and pollutant removal.
- Stable evaporation (2.04 kg m⁻² h⁻¹) with 96.5% rhodamine B degradation in 60 min.

**Supplementary Information:**

The online version contains supplementary material available at 10.1007/s40820-026-02206-8.

## Graphical abstract

**Figure:**
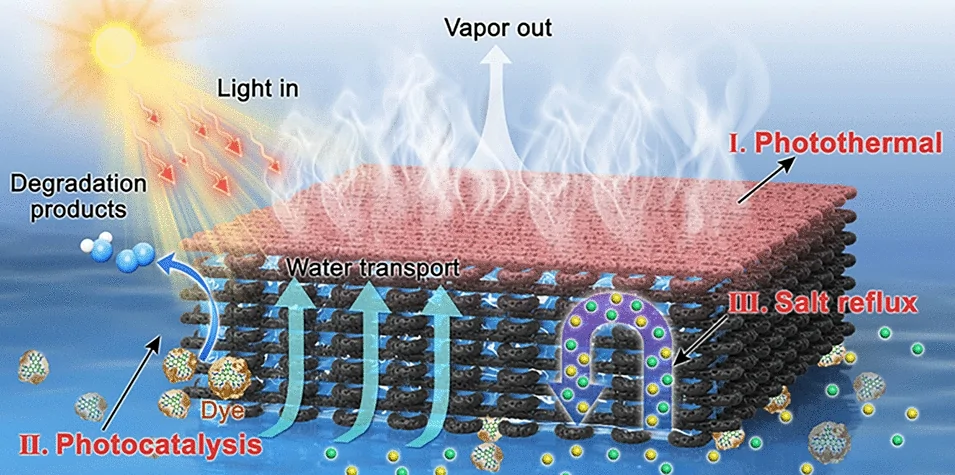

## Introduction

With the rapid advancement of society and industrialization, the worldwide water scarcity issue is becoming increasingly severe, and the demand for clean water is continuously increasing [1]. Desalination of seawater and treatment of complex industrial wastewater are effective strategies for alleviating the scarcity [2–5]. However, the widely used water treatment technologies, such as distillation, reverse osmosis, and nanofiltration, are limited by their complex equipment, high costs, and significant energy consumption, thereby restricting their application in remote areas [6]. Interfacial solar-driven water evaporation technology presents a sustainable method of using solar energy to produce clean water and holds great potential [7–12]. To improve the efficiency of interfacial water evaporation, researchers are currently focusing on the use of photothermal materials, optimization of water transport, and thermal management for enhanced energy efficiency [13–15]. Nevertheless, its practical deployment for complex industrial wastewater faces two coupled bottlenecks: High salinity causes salt crystallization that clogs the evaporator, while diverse organic pollutants foul the system and compromise water quality [16–20].

To tackle organic contamination, the integration of photocatalysts (e.g., as metal oxides, graphene-based materials, metal–organic frameworks) into solar evaporators has been explored, enabling simultaneous steam generation and pollutant degradation [21–27]. Among them, the porphyrin-based metal–organic framework PCN-224 exhibits exceptional broad-spectrum solar absorption and proven efficacy in degrading diverse refractory pollutants, including dyes and pharmaceuticals [28–34]. Moreover, we therefore hypothesize that a strategic combination of PCN-224 with a photothermal material like carbon black (CB) could yield a synergistic system, where PCN-224 functions not only as a potent photocatalyst but also as a photothermal enhancer for improved energy conversion. However, effectively incorporating and stabilizing photocatalytic components within a porous evaporator structure without compromising water transport or light absorption remains a key design challenge.

Simultaneously, salt accumulation during the evaporation of high-salinity feeds leads to blocked pores and reduced evaporation efficiency [35]. Several strategies have been developed to enhance salt resistance in evaporators, including local crystallization [36, 37], ion rejection [38, 39], Janus design [40, 41], and back-diffusion [42–46]. Of these, the back-diffusion strategy, which facilitates the continuous return of ions to the bulk solution, offers a fundamental solution to prevent salt accumulation. Previous studies have demonstrated that building vertically aligned structures in an evaporator can accelerate ion diffusion through directional confinement of the water path [47–50]. In this regard, 3D printing technology offers a powerful tool for fabricating hydrogel evaporators with precisely designed architectures. These engineered scaffolds, featuring interconnected macro-/micropores, not only promote excellent salt rejection and maintain high evaporation rates [51, 52] but also provide an ideal platform for the stable incorporation of multifunctional materials, such as photocatalysts, paving the way for devices that concurrently address both salinity and contamination.

**Fig. 1:**
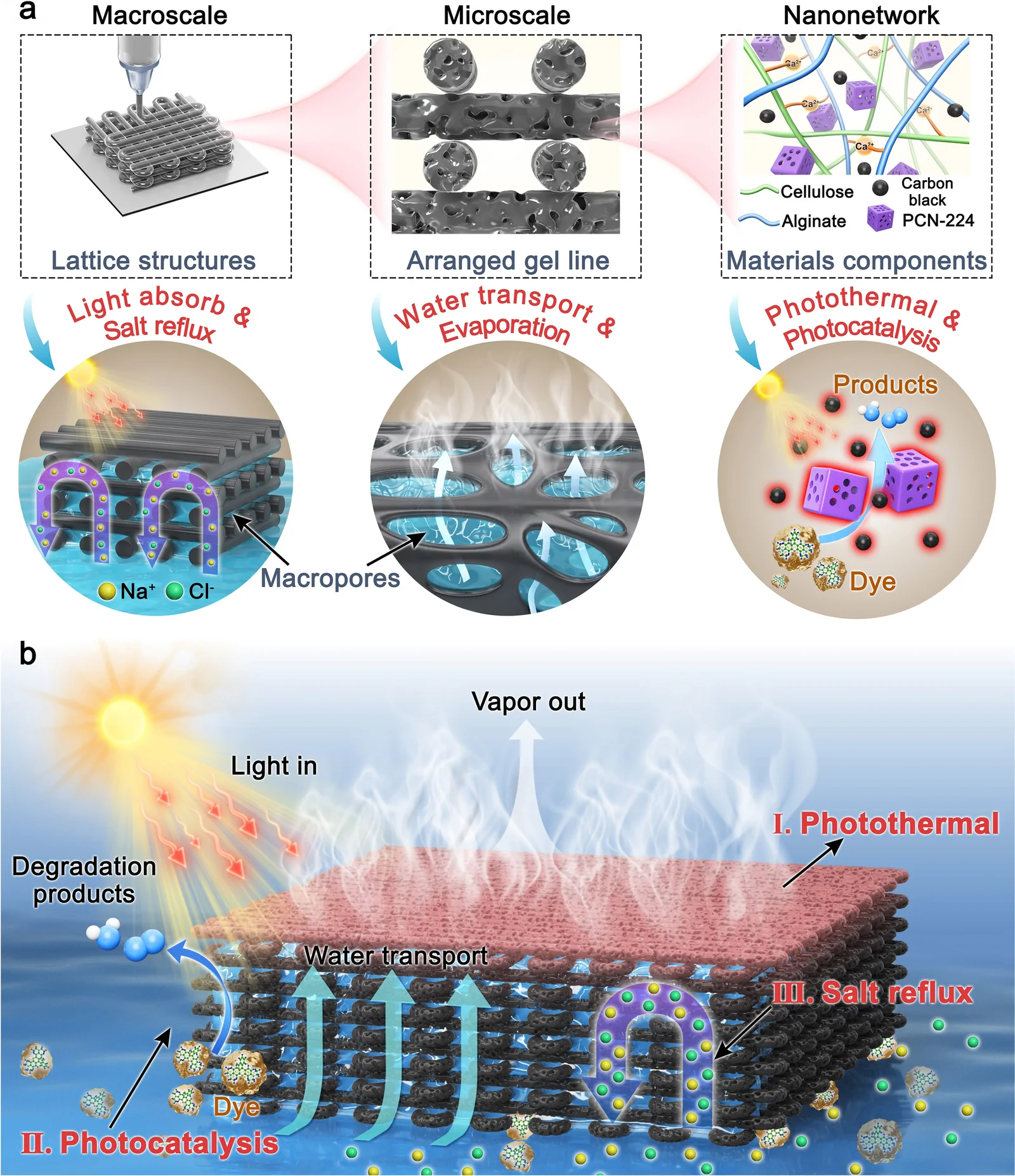
**a** Water evaporation, salt rejection, and photocatalytic properties of the PCN-224/CB@NFC/SA hydrogel scaffold. **b** Structure and function of the PCN-224/CB@NFC/SA hydrogel scaffold

Herein, we report a multifunctional 3D-printed photothermal hydrogel evaporator, engineered through rational material hybridization and structural design. The composite matrix integrates natural polysaccharides (sodium alginate, SA) with nanofibrillated cellulose (NFC) for reinforcement, CB nanoparticles for photothermal conversion, and PCN-224 for photocatalysis, forming a multi-cross-linked network via ionic and hydrogen bonds. This composite, denoted as PCN-224/CB@NFC/SA, is structured into a 3D-printed grid architecture that creates hierarchical fluidic channels, facilitating simultaneous rapid water transport and ion back-diffusion (Fig. 1a). Meanwhile, the evaporation process concentrates pollutants near the catalytic sites, thereby promoting the degradation reaction. The PCN-224/CB@NFC/SA achieves a high evaporation rate of 2.04 kg m^−2^ h^−1^ under one-sun illumination, which remains stable at 2.03 kg m^−2^ h^−1^ even in 3.5 wt% brine. Moreover, under a higher light intensity of 1.5 kW m^−2^, it exhibits exceptional photocatalytic activity, degrading 96.5% of rhodamine B (RhB) within 60 min. As illustrated in Fig. 1b, this multifunctional evaporator thus presents a promising solution for efficient treatment of high-salinity wastewater, enabling simultaneous clean water production and pollutant degradation.

## Experimental Section

### Materials

Zirconyl chloride octahydrate (ZrOCl_2_·8H_2_O, 99%) and meso-tetra (4-carboxyphenyl) porphine (97%) were purchased from Bide Pharmatech Co., Ltd. N, N-Dimethylformamide (DMF, AR, 99.5%), acetic acid (≥ 99%), ethanol (AR, ≥ 95%), sodium alginate (SA, RG, viscosity: 200-500 mpa.s), glycerol (99%), calcium chloride (CaCl_2_, AR, 96%), and rhodamine B (AR) were bought from Shanghai Aladdin Biochemical Technology Co., Ltd. Nanofibrillated cellulose (NFC, technical grade) was provided by Tianjin Wood Wizard Biotech Co., Ltd. Carbon black (CB, technical grade) was obtained from Tianjin Tianyi Century Chemical Products Technology Development Co., Ltd. Real dyeing wastewater (technical grade) from Jiangsu Shenghong Printing and Dyeing Co., Ltd.

### Preparation of a Porphyrinic Metal–Organic Framework (PCN-224)

For the synthesis of PCN-224, 1.2 g ZrOCl_2_·8H_2_O was initially dissolved in N, N-dimethylformamide (DMF, 500 mL) and subjected to ultrasonication for five min. Subsequently, 0.25 g meso-tetra(4-carboxyphenyl) porphine was introduced into the solution, followed by five min of ultrasonication. After the addition of 125 mL of acetic acid, the mixture was evenly distributed between two 500 mL Duran bottles and heated at 65 °C in a forced-air oven for 72 h. The precipitate formed was collected via centrifugation, washed with methanol, and dried under a vacuum at 80 °C.

### Ink Preparation

In this study, four inks were prepared based on a nanocellulose/sodium alginate (NFC/SA) matrix: (i) pure NFC/SA; (ii) PCN-224@NFC/SA with incorporated metal–organic framework (PCN-224); (iii) CB@NFC/SA blended with carbon black (CB); and (iv) a composite of PCN-224 and CB in NFC/SA (PCN-224/CB@NFC/SA).

The preparation process for NFC/SA ink is as follows: Firstly, 20 g of NFC solution (1.85 wt%) was mixed with 3 wt% of SA, homogenized at 10,000 rpm for 30 min, and magnetically stirred at 60 °C for 30 min. For the preparation of the PCN-224@NFC/SA ink, 2.5 mg g^−1^ of PCN-224 was added to the NFC/SA ink base. In the case of CB@NFC/SA ink preparation, PCN-224 in NFC/SA ink was replaced with an equivalent amount of CB. Lastly, for the preparation of PCN-224/CB@NFC/SA ink, both PCN-224 and CB solution (2.5 mg g^−1^ each) were simultaneously added to NFC/SA ink. The optimized loading of 2.5 mg g^−1^ for both CB and PCN-224 ensures a balance between performance and processability. This concentration of CB achieves a light absorption plateau of ~ 96%, while the 1:1 mass ratio provides the ideal rheological properties and yield stress required for high-precision 3D printing without structural collapse (Fig. S1a, b).

### 3D Printing

The ink was loaded into a 10-cc glue dispensing syringe and centrifuged for 5 min for defoaming. The CAD printing files were uploaded to a computer. The ink was printed through a syringe nozzle with a diameter of 0.55 mm at a printing speed of 10 mm s^−1^, driven by an air pressure of 30 psi. After printing, the printed frames were transferred into a 2 wt% CaCl_2_ bath for cross-linking for 1 h and then frozen in the refrigerator for 24 h.

## Results and Discussion

### Morphologies and Components of the Hydrogel Evaporation

**Fig. 2:**
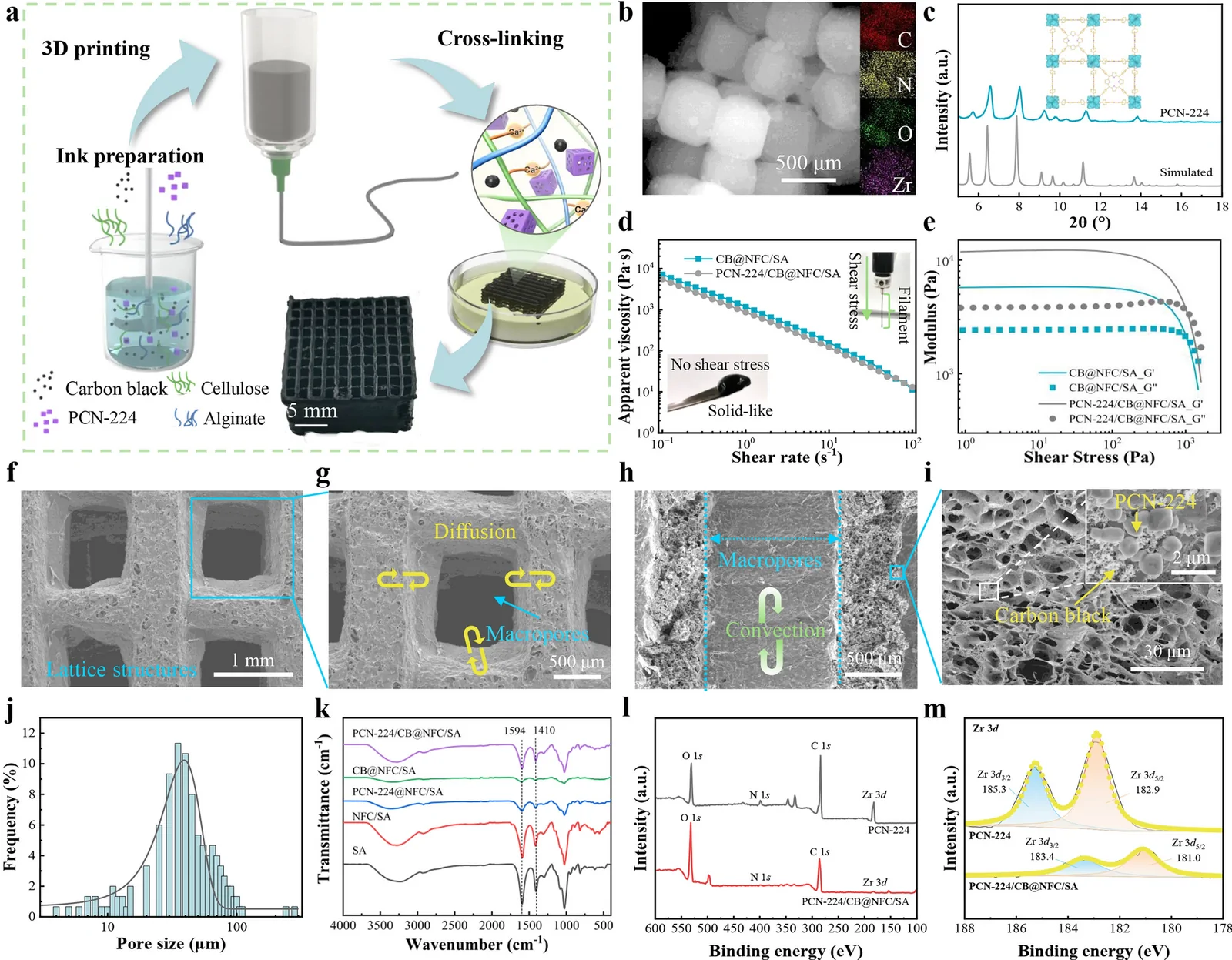
Characterizations of the lattice-like structure hydrogel evaporator. **a** Process for the preparation of hydrogel frames. **b** SEM image and EDS images of PCN-224. **c** XRD patterns of PCN-224. **d** Shear-thinning behavior of CB@NFC/SA ink and PCN-224/CB@NFC/SA ink (insert images showing inks with and without shear stress). **e** Storage modulus (G′) and loss modulus (G″) as a function of shear stress for the CB@NFC/SA ink and PCN-224/CB@NFC/SA ink. SEM images of a lattice structure: **f** top view of the PCN-224/CB@NFC/SA printed structure, **g** magnified top view, and **h** vertical section morphology. **i** SEM image showing the longitudinal micrometer-scale pore structure of PCN-224/CB@NFC/SA. The inset in **i** shows the morphology of PCN-224 and carbon black particles in PCN-224/CB@NFC/SA. **j** Pore size distribution of PCN-224/CB@NFC/SA.** k** FTIR spectra of SA, NFC/SA, PCN-224@NFC/SA, CB@NFC/SA, PCN-224/CB@NFC/SA.** l** XPS survey spectrum of the PCN-224 and PCN-224/CB@NFC/SA. **m** XPS Zr 3*d* spectra and their fitting peaks of the PCN-224 and PCN-224/CB@NFC/SA

Recent studies have emphasized that rational pore structure design in evaporator substrates is critical for enabling efficient water transport and brine return [49, 52, 53]. The direct ink writing (DIW) 3D printing technology can be used to build vertically aligned millimeter-scale grid structures (Movie S1). The ink was formulated with sodium alginate (SA) as the matrix, nanofibrillated cellulose (NFC) as a reinforcement, carbon black (CB) for photothermal conversion, and the metal–organic framework PCN-224 for photocatalysis (Fig. 2a). The synthesized PCN-224, characterized by a cubic morphology (~ 526 nm, Figs. 2b and S2) and a high specific surface area (1821 m^2^ g^−1^, Fig. S3), provides the active sites for pollutant degradation. EDS mapping confirmed the uniform distribution of C, N, O, and Zr elements (Fig. 2b inset). Furthermore, its phase purity was confirmed by XRD, showing a pattern consistent with the simulated structure (Fig. 2c). In addition, the band gap of PCN-224 was determined to be 2.96 eV (Fig. S4), indicating its potential for visible light-driven photocatalysis.

The printability of the ink is paramount for constructing stable 3D architectures. A composite ink, denoted as PCN-224/CB@NFC/SA, was formulated by homogeneously dispersing nanoscale CB and PCN-224 within a gel matrix of 1.85 wt% NFC and 3 wt% SA (Fig. S5). This uniformity and high viscosity are achieved through physical entanglements and hydrogen bonding between the components [54]. Rheological evaluation confirmed its excellent printability. All ink formulations exhibited solid-like behavior and significant shear thinning (Figs. 2d and S6a), enabling smooth extrusion. This was further verified by a storage modulus (G′) substantially higher than the loss modulus (G″) (Figs. 2e and S6b). Specifically, the PCN-224/CB@NFC/SA ink showed a G′ plateau of ~ 8249 Pa and a yield stress of ~ 344 Pa, indicating robust shape retention and the ability to form spanning structures [55, 56]. Using this ink, we successfully printed a 20 × 20 × 10 mm^3^ grid (Fig. S7).

Subsequent CaCl₂ cross-linking enhanced its mechanical integrity, yielding a hierarchically porous system whose porous structure was characterized by scanning electron microscopy (SEM). Top view morphology images (Fig. 2f, g) reveal macropores with dimensions of approximately 1 × 1 mm^2^ and printed fibers exhibiting a width of ~ 930 μm. The macroscopic pore structure facilitates timely water transport and enables rapid diffusion of salt ions [57, 58]. The cross-sectional view (Fig. 2h) confirms the highly vertical alignment of the grid, while higher-magnification images show that the scaffold is permeated with numerous pores/micrometer-scale pores, with sizes predominantly distributed between 20 and 80 μm (Fig. 2i, j). Importantly, the photothermal agent (CB) and photocatalytic particles (PCN-224) are uniformly distributed throughout the scaffold (Fig. 2i inset), which is favorable for simultaneous photothermal conversion and pollutant degradation.

The chemical structure of the hydrogel was verified by FTIR and XPS. The FTIR spectra (Fig. 2k) show the antisymmetric –COO– and symmetric –COO– peaks shifted from 1593 and 1406 cm^−1^ to 1594 and 1410 cm^−1^, confirming successful ionic cross-linking with Ca^2+^. XPS survey spectra (Fig. 2l) of the composite confirm the presence of C, N, O, and Zr, verifying the successful integration of PCN-224. In the high-resolution Zr 3*d* spectra (Fig. 2m), the peaks of pure PCN-224 at 185.3 eV (Zr 3*d*_3/2_) and 182.9 eV (Zr 3*d*_5/2_) shift to lower binding energies (183.4 and 181.0 eV) in the composite. This downward shift indicates an increase in electron density around Zr nodes, suggesting strong electronic interactions between the MOF and the NFC/SA matrix.

The PCN-224/CB@NFC/SA composite also demonstrated essential physical properties for application. It exhibited superhydrophilicity, with a water contact angle of zero within 0.365 s (Fig. S8). Swelling tests (Table S1) showed a minimal volume expansion (24% in water and only 5% in 10% NaCl brine), confirming excellent dimensional stability in saline environments. Furthermore, the framework showed exceptional cyclic compressibility, retaining 71.4% of its initial strength after 20 compression cycles at 50% strain, with consistent stress–strain hysteresis (Fig. S9).

### Heat Management and Water Evaporation Performance of the Hydrogel Evaporator

**Fig. 3:**
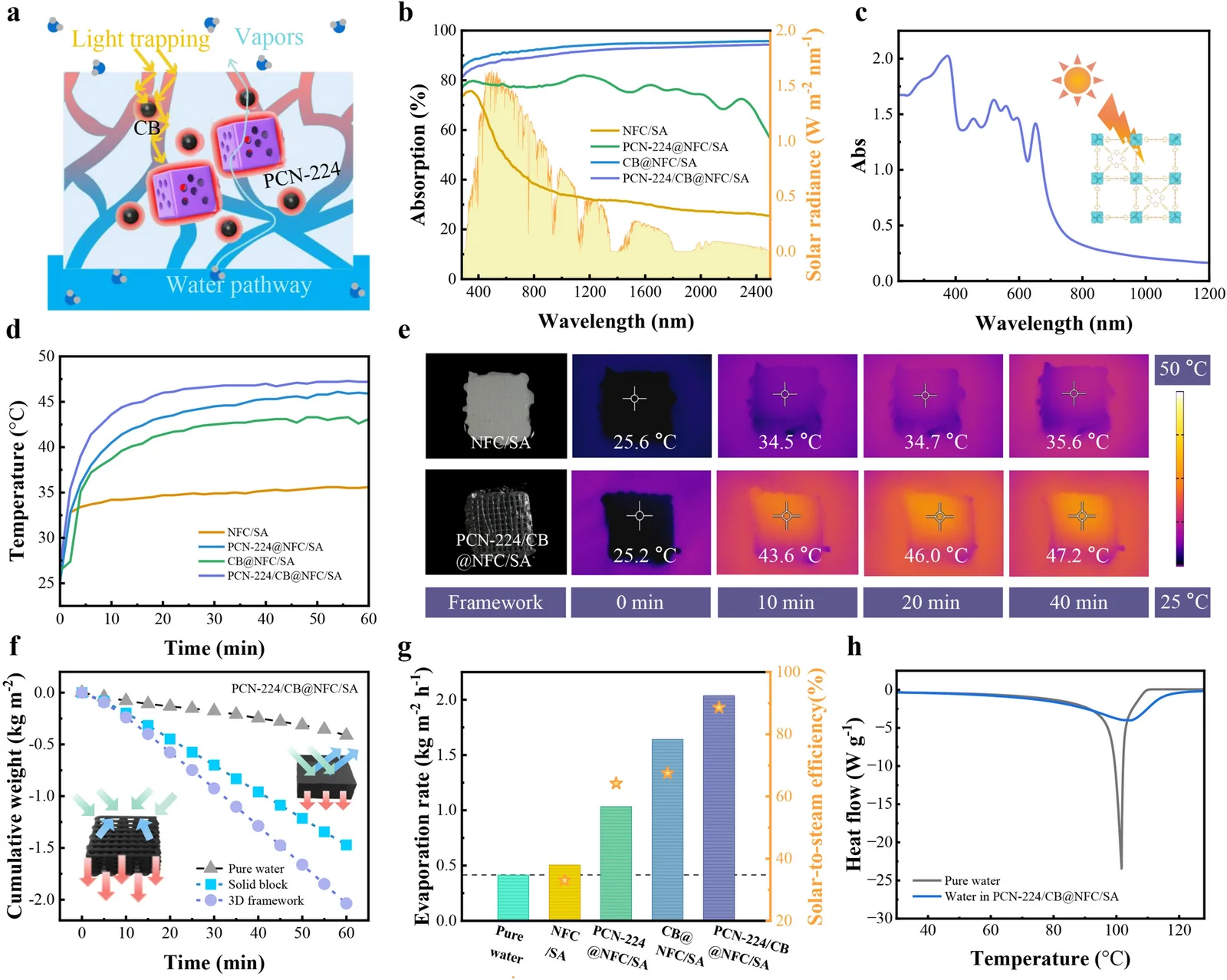
Characterization of thermal management and water evaporation performance of the lattice-like hydrogel evaporator. **a** Diagram of the light absorption and water evaporation mechanism of the hydrogel. **b** UV–Vis–NIR spectra of the evaporators and normalized spectral solar irradiance density of the air mass 1.5 global (AM 1.5 G) tilted solar spectrum. **c** UV–Vis–NIR spectra of PCN-224. **d** Surface temperature of NFC/SA, PCN-224@NFC/SA, CB@NFC/SA, and PCN-224/CB@NFC/SA as a function of the time of solar irradiation (1.0 kW m^−2^) over 60 min period. **e** Infrared thermal images show the surface temperature of NFC/SA and PCN-224/CB@NFC/SA at irradiation times of 0, 10, 20, and 40 min. **f** Mass change of pure water, solid block-shaped PCN-224/CB@NFC/SA, and 3D framework-shaped PCN-224/CB@NFC/SA evaporated over time. **g** Comparison graph of the evaporation rate and energy efficiency of pure water and four hydrogel evaporators. **h** DSC vaporization curves of pure water and water in PCN-224/CB@NFC/SA

Efficient light absorption is a prerequisite for high-performance hydrogel evaporators. In the PCN-224/CB@NFC/SA composite, carbon black (CB) functions as an efficient broadband photothermal material [59, 60]. Meanwhile, PCN-224 serves primarily as a visible light-harvesting photocatalyst for pollutant degradation and additionally as a photothermal enhancer (Fig. 3a). The synergy between these components not only improves evaporation efficiency through enhanced heat generation but also enables advanced oxidation processes for water purification [61, 62]. This synergy is fundamentally rooted in the strong electronic interaction and energy transfer between PCN-224 and CB. This is evidenced by the significantly enhanced light absorption, apparent band gap reduction, and quenched photoluminescence (PL) intensity of the composite compared to pristine PCN-224 (Fig. S10a-c). Specifically, the PL quenching suggests that CB acts as an electron sink for PCN-224, suppressing charge recombination, and the proposed charge transfer mechanism is illustrated in Fig. S11.

The light absorption capabilities of hydrogels with different components were systematically compared (Fig. 3b). The light absorption of CB@NFC/SA and PCN-224/CB@NFC/SA reached 95.7% and 94.0%, respectively, which are significantly higher than that of the PCN-224@NFC/SA (54.4%) and the pristine NFC/SA (29.9%). It is worth noting that PCN-224 itself exhibited strong visible light absorption (Fig. 3c). The marginal decrease in absorption of the composite compared to pure CB is attributed to interfacial light scattering between PCN-224 and CB (Fig. S12a, b).

Beyond efficient light harvesting, effective thermal management is also critical for achieving high-performance evaporation. The hydrogels maintained low thermal conductivities (0.071-0.084 W m⁻^1^ K⁻^1^), effectively minimizing parasitic heat loss to the underlying water (Fig. S13) [63–65]. Under simulated solar illumination in a floating configuration (Fig. S14), the steady-state temperatures of NFC/SA, PCN-224@NFC/SA, CB@NFC/SA, and PCN-224/CB@NFC/SA were 35.6, 43.1, 45.9, and 47.2 °C, respectively (Fig. 3d). The 1.3 °C temperature elevation in PCN-224/CB@NFC/SA over CB@NFC/SA specifically indicates that PCN-224 provides supplementary photothermal conversion. Infrared thermography analysis (Figs. 3e and S15) further validated this thermal localization, with PCN-224/CB@NFC/SA maintaining superior surface heating compared to the baseline NFC/SA.

The superior photothermal and structural properties of the PCN-224/CB composite directly translate to outstanding solar evaporation performance. Under 1 kW m^−2^ irradiation, the 3D-printed lattice-structured PCN-224/CB@NFC/SA demonstrated exceptional performance with an evaporation rate of 2.04 kg m^−2^ h^−1^ (Fig. 3f). This represents a 39% improvement over its solid block counterpart (1.47 kg m^−2^ h^−1^) and 4.98 times higher than the evaporation rate of pure water (0.41 kg m^−2^ h^−1^).

To further elucidate the structural advantages of the 3D-printed design, the macrostructure of 3D-printed lattices, particularly the line spacing (*z*), was optimized to maximize the evaporation rate [66]. According to boundary layer theory, vapor escape is governed by the boundary layer thickness (*δ*_b_), which can be expressed as Eq. (1):

1 $${\delta}_{b}=\frac{\left(z+D\right)\cdot ln(\frac{8(z+D/2)}{\pi D})}{2\pi }$$

where *D* is the fiber diameter.

To ensure unimpeded vapor diffusion, the spacing must satisfy the critical criterion *z* > 2*δ*_b_. As shown in Fig. S16a-c, for the sample with 1 mm initial spacing, the actual dried spacing (*z* ≈ 0.5 mm) is smaller than 2*δ*_b_, leading to boundary layer overlap and high diffusion resistance. Conversely, while a 3 mm spacing offers minimal resistance, the significantly reduced density of evaporative fibers limits the total active surface area. The optimized 2 mm spacing (actual *z* ≈ 1 mm after drying) satisfies *z* > 2*δ*_b_ (1000 µm > 854 µm), achieving an ideal balance between minimized diffusion resistance and maximized evaporative surface area. The 3D printing process demonstrated high manufacturing consistency (*z* = 1133 ± 95 µm), providing a sufficient buffer above the critical threshold to ensure stable performance despite minor geometric deviations (Fig. S17).

Beyond the structural optimization, the intrinsic contributions of individual components were also deconvoluted. The evaporation rates of CB@NFC/SA and PCN-224@NFC/SA were 1.64 and 1.03 kg m^−2^ h^−1^, respectively, both significantly higher than the NFC/SA substrate (0.50 kg m^−2^ h^−1^) (Fig. S18). This confirms that both CB and PCN-224 contribute to the photothermal effect. Most importantly, the PCN-224/CB composite outperformed the CB-only sample, providing direct evidence that the synergy between the two components, as previously indicated by the enhanced surface temperature, effectively boosts evaporation performance.

The enhanced evaporation kinetics are also underpinned by thermodynamic factors. Thermogravimetric analysis (Fig. S19) indicates facilitated water evaporation in the PCN-224/CB@NFC/SA composite compared with the other samples. Low-temperature differential scanning calorimetry (DSC) analysis reveals that this behavior is associated with a reduction in the evaporation enthalpy, which can be attributed to the nanoconfinement effect introduced by the microporous PCN-224 within the hydrogel matrix. This confinement facilitates the transition of bulk free water (FW) into intermediate water (IW) with weakened hydrogen bonding (Fig. S20). High-temperature DSC measurements further confirm that the equivalent evaporation enthalpy of water in the composite is lower than that of pure water (Fig. 3h).

Consequently, the optimized PCN-224/CB@NFC/SA lattice achieved a high solar-to-vapor conversion efficiency of 88.6% (Fig. 3g). The solar-to-steam conversion efficiencies of the other samples, such as NFC/SA, PCN-224@NFC/SA, and CB@NFC/SA, were 33.0%, 64.2%, and 67.5%, respectively. Furthermore, the 3D-printed lattice exhibited robust structural integrity and mechanical stability (87.9% strength retention) after wet–dry cycling and aging, ensuring long-term practical durability (Figs. S21 and S22). These results demonstrate that the PCN-224/CB@NFC/SA composite is an efficient hydrogel evaporator, successfully integrating component synergy (between PCN-224 and CB for enhanced light absorption and photothermal conversion) with structural ingenuity (through 3D printing for optimized vapor escape).

### Salt-Rejecting Performance

Salt crystallization during brine evaporation blocks light and impairs performance. A rational porous structure is key to mitigating this, as efficient longitudinal water transport ensures hydration while enhancing salt rejection [49, 58]. To demonstrate this, a comparison was made between the 3D-printed lattice evaporator (with well-aligned, low-tortuosity pores) and a solid block control (with disordered, high-tortuosity pores). Quantitative characterization (Table S2) reveals that the 3D framework and solid block exhibit tortuosities of 1.25 and 6.29, respectively, resulting in significantly lower ion transport resistance (300 Ω compared to 5000 Ω, Fig. S23). This streamlined architecture minimizes the diffusion path, facilitating rapid salt back-diffusion.

**Fig. 4:**
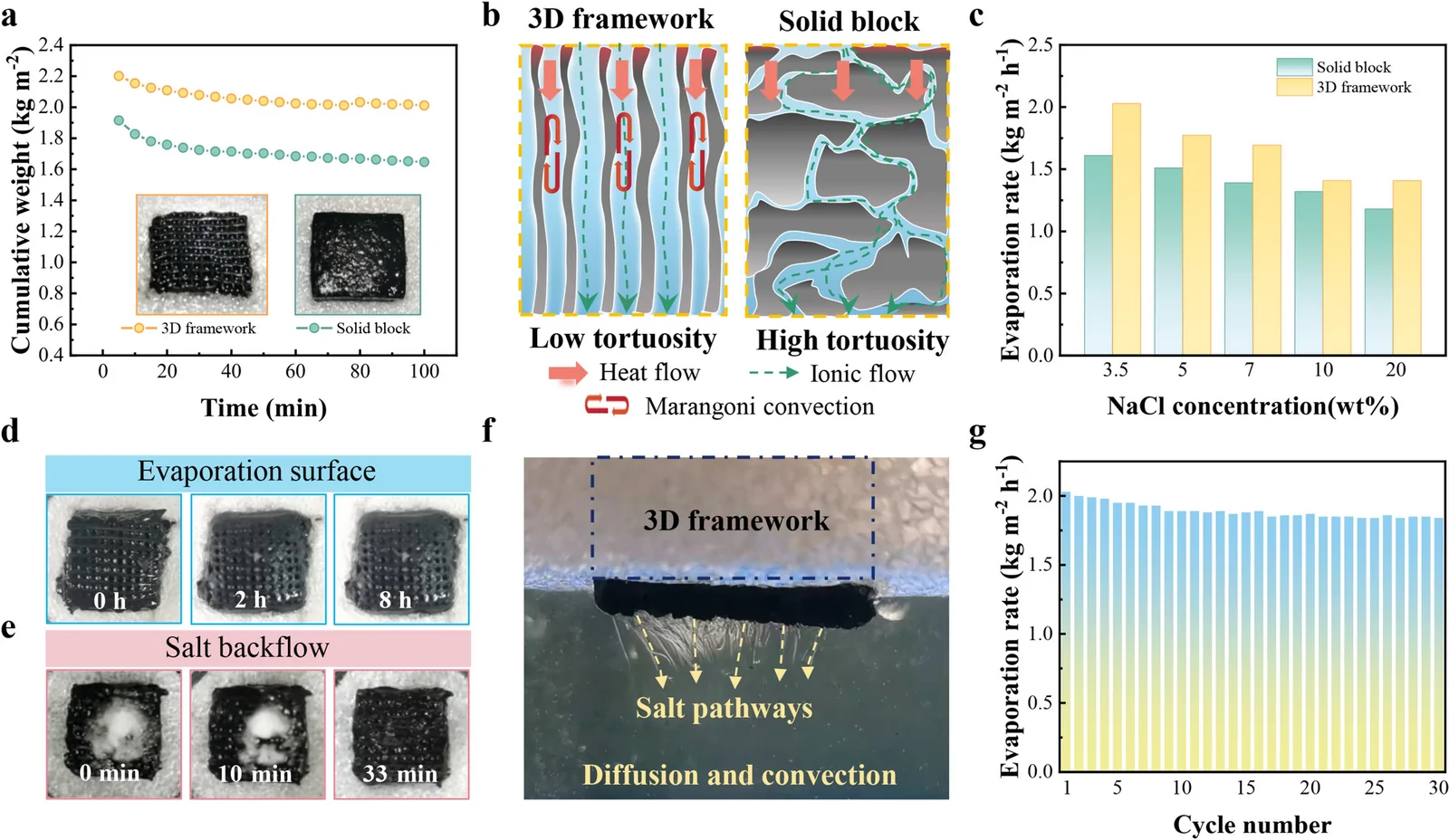
Salt-rejecting performance of the hydrogel evaporator. **a** Evaporation rate and top view morphology pictures of solid block PCN-224/CB@NFC/SA and 3D framework PCN-224/CB@NFC/SA in a 3.5 wt% NaCl solution. **b** Schematic illustration of the salt rejection mechanisms in different structures. **c** Evaporation rate of solid block-shaped PCN-224/CB@NFC/SA and 3D framework-shaped PCN-224/CB@NFC/SA in various brine salinity under 1 kW m.^−2^ irradiation.** d** Digital images of the hydrogel evaporator after 8 h of evaporation. **e** Salt-rejecting performance of the hydrogel evaporator. **f** Images of the phenomenon of salt reflux in a 3D framework PCN-224/CB@NFC/SA.** g** Cyclic evaporation performance of PCN-224/CB@NFC/SA in 3.5% NaCl (1 h per cycle)

Within a 3.5 wt% NaCl solution, the 3D framework PCN-224/CB@NFC/SA consistently exhibited higher evaporation rates than the block-shaped control (Fig. 4a). The uniformly arranged pores facilitate optimal water supply and salt reflux, while the winding pores within the solid mass trap salt ions, hindering salt reflux (Fig. 4b). Critically, this salt rejection is not merely governed by passive diffusion, but is likely assisted by Marangoni convection, which is induced by the vertical temperature gradient and salinity gradient [44–46]. This effect arises from the significant vertical temperature gradient (10.3 °C) (Fig. S24a) and the evaporation-induced salinity gradient, the latter of which was confirmed by electrical impedance tomography (EIT) (Fig. S24b-e).

To further verify the effect of structure on salt rejection performance, the evaporation rate of both the solid block PCN-224/CB@NFC/SA and the 3D framework PCN-224/CB@NFC/SA was measured under one sun in various brine salinities (3.5 to 20 wt% NaCl). The 3D framework consistently outperformed the solid block at all salinity levels (Fig. 4c). For the 3D framework PCN-224/CB@NFC/SA, the evaporation rates were 2.03, 1.77, 1.69, 1.43, and 1.43 kg m^−2^ h^−1^ when immersed in 3.5, 5, 7, 10, and 20 wt% NaCl solutions, respectively. In contrast, the solid block PCN-224/CB@NFC/SA exhibited evaporation rates of 1.58, 1.53, 1.52, 1.34, and 1.31 kg m^−2^ h^−1^ under the same salinity conditions. The observed decline in evaporation rate at higher salinities, relative to pure water, can be attributed to the reduced water activity and increased evaporation enthalpy at higher salinity [67]. These results demonstrate that the aligned, low-tortuosity pores in the 3D framework facilitate efficient salt back-diffusion.

To demonstrate the saltwater evaporation performance of the 3D framework PCN-224/CB@NFC/SA more intuitively, it was immersed in 3.5 wt% brine for 8 h of light exposure. Notably, no significant salt accumulation was observed on the surface (Fig. 4d). Further, to assess the salt diffusion capability, 0.5 g solid NaCl was placed on the top surface of the 3D framework PCN-224/CB@NFC/SA and tested under irradiation at 1 kW m^−2^. The solid salt was gradually dissolved after 33 min (Fig. 4e), and traces of salt diffused from the 3D framework back into the water (Fig. 4f). The salt transport flux measurements (Test S8 and Fig. S25), which showed a flux of 4.125 kg m^−2^ h^−1^ for the 3D-printed framework, significantly higher than the 0.75 kg m^−2^ h^−1^ for the solid block, confirm more efficient salt reflux in the aligned, low-tortuosity structure. To evaluate robustness under extreme conditions, a staircase salt-loading test was conducted. Despite the bulk salinity reaching 13.4 wt%, the 3D framework exhibited a responsive salt flux that rebounded to 2.25–3.00 kg m⁻^2^ h⁻^1^ upon successive salt additions (Table S3), proving the architecture effectively prevents saturation via a gradient-driven mechanism. Consequently, the 3D grid structure outperformed the block control and maintained high evaporation rates over 30 cycles (Fig. 4g).

### Organic Pollutant Degradation Capability of the Hydrogel Evaporator

The degradation efficiency of pollutants is critically influenced by photothermal conversion-induced temperature variations and saline conditions. The CB@NFC/SA (without PCN-224) and PCN-224/CB@NFC/SA (with PCN-224) were used for photodegradation test. Among these, PCN-224 has been demonstrated to possess both rapid adsorption properties (Fig. S26a, b and Table S4) and significant photocatalytic activity, effectively degrading RhB under illumination (Fig. S27a-c).

**Fig. 5:**
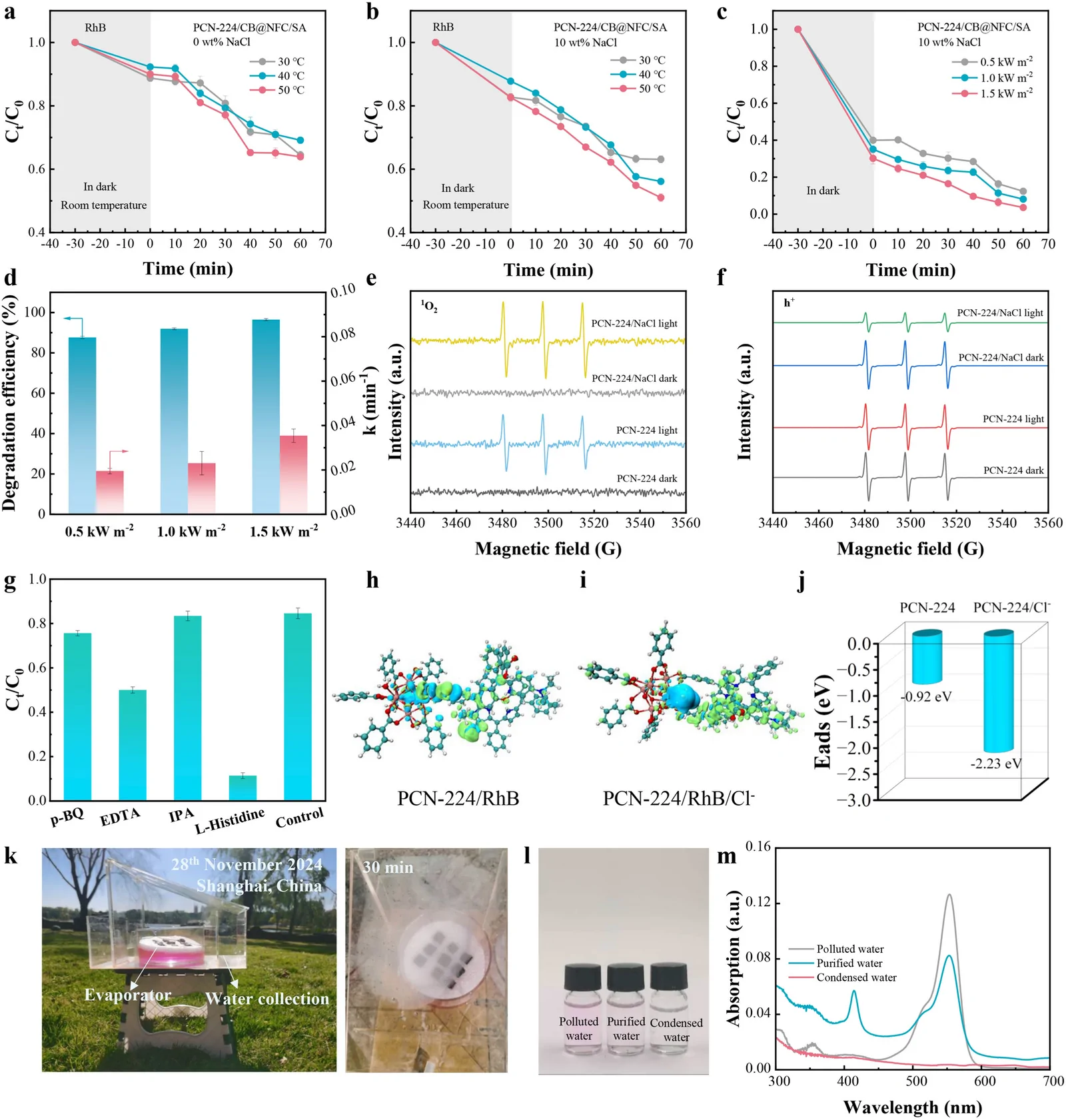
Effect of water temperature on the degradation rate of RhB catalyzed by PCN-224/CB@NFC/SA under one-sun irradiation: **a** in 0 wt% NaCl solution under different water temperatures and **b** in a 10 wt% NaCl solution under different water temperatures. **c** Degradation of RhB (with 10 wt% NaCl) by PCN-224/CB@NFC/SA under different light intensities. **d** Degradation efficiency and photocatalytic rate of RhB (with 10 wt% NaCl) by PCN-224/CB@NFC/SA under different light intensities at a water temperature of 50 °C. EPR spectra of reactive species over PCN-224 under saline (10 wt% NaCl) and salt-free conditions after light irradiation for 10 min: **e** TEMP-^1^O_2_ in aqueous solution. **f** TEMPO for hole (h^+^) detection in aqueous solution. **g** Effect of different quenchers on photocatalytic degradation efficiency. Difference charge density of RhB on** h** PCN-224 and **i** PCN-224/Cl, where green and blue represent electron depletion and electron accumulation, respectively. **j** Calculated adsorption energies. **k** Schematic illustration of the experimental setup for evaluating clean water generation performance. **l** Pictures of polluted water, purified water, and condensed clean water. **m** Absorption spectra of polluted water, purified water, and a condensed clean water

The experimental results revealed a critical difference between the two materials. For CB@NFC/SA (Fig. S28a), the RhB concentration decreased due to adsorption at room temperature. However, when exposed to illumination under controlled water temperatures, the concentration rebounded to its original level within 60 min. This phenomenon suggests that CB@NFC/SA primarily undergoes physical adsorption, where the increased molecular kinetic energy at elevated temperatures triggers the endothermic desorption of dye molecules. In contrast, PCN-224/CB@NFC/SA exhibited superior degradation performance across all temperatures (30, 40, and 50 °C), and achieved 36.1% efficiency at 50 °C (Fig. 5a), rather than mere physical adsorption.

Notably, this performance advantage was significantly amplified under high-salinity conditions. In simulated hypersaline wastewater (10 wt% NaCl), CB@NFC/SA showed negligible adsorption or degradation of RhB (Fig. S28b). Conversely, NaCl remarkably improved the catalytic efficiency of PCN-224/CB@NFC/SA, with degradation reaching 50.0% at 50 °C (Fig. 5b). This enhancement, coupled with maintained structural integrity (Fig. S29), underscores the excellent stability and salt promotion effect of the PCN-224-integrated system. Control experiments confirmed that RhB is resistant to self-photolysis (Fig. S30), validating the indispensable role of the PCN-224 photocatalyst.

Based on these optimized temperature and salinity parameters, the impact of solar irradiance (0.5, 1.0, and 1.5 kW m^−2^) was investigated at 10 wt% NaCl and 50 °C. As shown in Fig. 5c, d, both the degradation efficiency and the apparent rate constant increased with increasing light intensity. The degradation efficiencies reached 87.6%, 91.9%, and 96.5%, with corresponding rate constants of 0.0195, 0.02305, and 0.03543 min⁻^1^, respectively. The system maintained over 92.2% efficiency after ten cycles (Figs. S31 and S32) and showed a stable 12-h evaporation rate (~ 1.40 kg m^−2^ h^−1^) in 10% NaCl/RhB solution (Fig. S33). Posttest SEM and TG/DTG profiles (Figs. S34 and S35) further confirmed its structural durability and antifouling capability. Notably, while the system effectively degraded methylene blue (MB), its performance was partially inhibited by high salinity (Fig. S36a, b). This dye-specific behavior suggests a degradation pathway influenced by molecular charge and structure, which aligns with the charge-mediated mechanism discussed below.

To elucidate the enhancement mechanism, the possible generation of reactive species (·OH, ·O₂⁻, ^1^O₂, and h⁺) was probed using electron paramagnetic resonance (EPR) spectroscopy under both saline and salt-free conditions (Figs. S37a, b and 5e, f). The ROS signals under saline conditions are stronger than those under salt-free conditions, indicating that the presence of Cl⁻ promotes the generation of reactive oxygen species. Meanwhile, the weakened TEMPO signal under saline conditions suggests that photogenerated h⁺ is more rapidly consumed, possibly due to the oxidation of Cl⁻ by h⁺. Radical quenching experiments (Fig. 5g) showed that the addition of IPA (·OH scavenger) and p-BQ (·O_2_^−^ scavenger) only marginally reduced the efficiency to 83.4% and 75.6%, respectively (control: 84.6%). In contrast, the introduction of L-histidine (a ^1^O₂ scavenger) caused a dramatic drop in efficiency to 11.41%, indicating that ^1^O₂ is the dominant reactive oxygen species in the photocatalytic process. Furthermore, the degradation efficiency decreased to 50.0% upon the addition of EDTA, suggesting that photogenerated holes (h^+^) also participate in the oxidation pathway. Although the transient nature of reactive chlorine species (RCS) makes them difficult to directly capture via EPR in aqueous systems, the pronounced attenuation of the TEMPO signal (Fig. 5f), together with the enhanced degradation performance observed under saline conditions, suggests the possible involvement of Cl⁻-mediated hole transfer processes. These results indicate that Cl⁻ may act as a hole trapping and charge transfer mediator, facilitating charge separation and promoting the generation of ^1^O₂, thereby enhancing the photocatalytic degradation of RhB [68–70]. In addition, higher sunlight intensity provides greater excitation energy, which improves electron–hole separation in PCN-224/CB@NFC/SA and consequently increases the degradation efficiency.

In addition to the chemical pathway discussed above, surface interaction effects were further investigated. Zeta potential analysis (Fig. S38) further supports the salt-promoted interaction between RhB and the catalyst surface. The mixture exhibits a more negative zeta potential in 10 wt% NaCl (− 20.63 mV) than under salt-free conditions (− 4.87 mV), indicating a stronger electrostatic interaction between RhB and the catalyst surface [71]. To theoretically validate the salt promotion effect, density functional theory (DFT) calculations were performed (Test S15). The charge density difference (CDD) maps (Fig. 5h, i) reveal evident interfacial charge redistribution upon RhB adsorption. The blue and green isosurfaces represent electron accumulation and electron depletion, respectively. In the PCN-224/RhB system (Fig. 5h), electron depletion is mainly observed around the porphyrin framework of PCN-224, while electron accumulation appears on the RhB molecule, indicating charge transfer from the catalyst surface to the adsorbate. After introducing Cl⁻ (Fig. 5i), the electron accumulation around the RhB molecule becomes more pronounced, accompanied by stronger electron depletion on the PCN-224 framework, suggesting enhanced interfacial charge transfer. AIM charge analysis further confirms this behavior, showing that the charge transfer from PCN-224 to RhB increases from 0.402 to 0.732 e after the introduction of Cl⁻. Meanwhile, the adsorption energy decreases from − 0.92 to − 2.23 eV, indicating a significantly stronger adsorption interaction between RhB and the catalyst surface (Fig. 5j). These results suggest that Cl⁻ enhances both interfacial charge transfer and adsorption strength, which contributes to the improved photocatalytic performance under saline conditions.

Finally, the integration of PCN-224/CB@NFC/SA with a condensate collection device harnesses solar energy to achieve the dual functions of pollutant degradation and clean water production. The material rests on the surface of the polluted liquid, where it absorbs solar energy to induce water evaporation, and the vapor condenses to liquid water in the transparent top layer and is collected by gravity (Fig. 5k). After 4 h of light exposure, the chromaticity of the treated water was significantly reduced, the condensate was colorless and transparent (Fig. 5l), and the UV spectra showed that the RhB characteristic peak of the condensate completely disappeared (Fig. 5m). In practical textile wastewater treatment, the COD dropped from 1828 to 25 ppm (Fig. S39), with excellent stability over 5 cycles (evaporation rate ~ 1.74 kg m⁻^2^ h⁻^1^, Fig. S40). Analyses confirmed the total removal of proteinaceous/humic substances (Fig. S41) and heavy metals; specifically, Sb was removed from 8.09 mg L⁻^1^ to below detection limits (Table S5), while chromaticity fell from 226 Hazen to undetectable levels (Fig. S42). With a low production cost (~ 91.5 $ m^−2^, Tables S6 and S7), this 3D-printed evaporator outperforms reported materials (Table S8) by combining a high evaporation rate (2.04 kg m⁻^2^ h⁻^1^) with synergistic photocatalysis and robust salt resistance for efficient industrial wastewater purification.

## Conclusions

In summary, we have successfully designed and fabricated a multifunctional 3D-printed hydrogel evaporator (PCN-224/CB@NFC/SA) that integrates photothermal evaporation, salt resistance, and photocatalytic degradation into a single platform. The vertically aligned grid structure, realized via direct ink writing, facilitates efficient water transport and salt back-diffusion, effectively suppressing salt crystallization. Utilizing a dual-functional photothermal–photocatalytic system composed of carbon black and PCN-224, this evaporator achieves a high evaporation rate of 2.04 kg m^−2^ h^−1^ under one-sun illumination and demonstrates exceptional photocatalytic activity that degrades 96.5% of RhB within 60 min under 1.5 kW m^−2^ light irradiation. More importantly, the system effectively treats real dyeing wastewater, significantly reducing its COD and removing organic contaminants from the condensate. This work offers a scalable and environmentally friendly strategy for solar-driven water production and purification, presenting a promising solution for tackling complex industrial wastewater challenges.

## Supplementary Information

Below is the link to the electronic supplementary material.

Supplementary file1 (DOCX 5088 KB)

Supplementary file2 (MP4 10224 KB)
