# Supplementary material for: Hierarchically Architected 3D-Printed Hydrogel Evaporators Enable Synergistic Salt Management and Photocatalytic Purification
Source: Nanomicro Lett. 2026 May 11;18:363. doi: 10.1007/s40820-026-02206-8 (PMC13161444; doi:10.1007/s40820-026-02206-8)
Supplement: Supplementary file 1 — Supplementary file1 (DOCX 5088 KB) [file 40820_2026_2206_MOESM1_ESM.docx]

Supporting Information for

**Hierarchically Architected 3D-Printed Hydrogel Evaporators Enable Synergistic Salt Management and Photocatalytic Purification**

Xin Yang^1^, Xinqi Guo^1^, Yankuan Tian^3^, Rong Zhou^4^, Yifei Gong^1^, Chengming Zhang^1^, Feng Ji^1^, Liu Liu^2^, Faxue Li^1 *^, Ruiyun Zhang^1 *^, Jianyong Yu^2^, Tingting Gao^1 *^

^1^ Key Laboratory of Textile Science & Technology, Ministry of Education, College of Textiles, Donghua University, Shanghai 201620, P. R. China

^2^ Innovation Center for Textile Science and Technology, Donghua University, Shanghai 201620, P. R. China

^3^ Key Laboratory of Multifunctional Nanomaterials and Smart Systems, Suzhou Institute of Nano-Tech and Nano-Bionics, Chinese Academy of Sciences, Suzhou 215123, P. R. China

^4^ College of Textiles and Fashion, Hunan Institute of Engineering, Xiangtan, 411104, P. R. China

* Corresponding authors. Email: [gaott@dhu.edu.cn](mailto:gaott@dhu.edu.cn) (Tingting Gao), [ryzhang@dhu.edu.cn](mailto:ryzhang@dhu.edu.cn) (Ruiyun Zhang), [fxlee@dhu.edu.cn](mailto:fxlee@dhu.edu.cn) (Faxue Li)

**Test S1 Solar steam generation experiment**

Indoor sunlight was generated using a xenon lamp. The solar steam generator experiment was carried out by placing the hydrogel framework in a container, which was fixed in foam and connected to the water body. The sample was illuminated under simulated conditions in the dark and under sunlight with a radiation intensity of 1 kW m^−2^ to characterize water evaporation of the system [S1, S2]. The evaporator device was placed on an electronic scale to measure the weight loss of water in real time. Thermocouples and an infrared thermal imager were used to record the temperature of the samples. The ambient temperature and humidity were approximately 25 °C and 50%, respectively.

The evaporation rate ($\dot{\text{m}}$) was calculated by the equation (S1):

|  | $\dot{\text{m}}\text{= }\frac{\text{dm}}{\text{S}\text{ }\text{×}\text{ }\text{dt}}$ | (S1) |
| --- | --- | --- |

Among them, *m* is the mass loss of the liquid during photoevaporation, *t* is the illumination time, and *S* is the surface area of the sample.

The solar-to-steam efficiency $\text{η}$ in the light-to-steam conversion process can be calculated by equation (S2):

|  | $\text{η}\text{=}\frac{\dot{\text{m}}\text{h}_{\text{L}\text{V}}}{\text{P}_{\text{in}}}$ | (S2) |
| --- | --- | --- |

In the equation, $\dot{\text{m}}$ is the net evaporation rate and calculated as the total evaporation rate minus the dark evaporation rate, $\text{h}_{\text{LV}}$ is the total enthalpy, including phase change enthalpy and sensible heat enthalpy, and $\text{P}_{\text{in}}$is the total solar energy input.

The total $\text{h}_{\text{LV}}$ includes the phase change enthalpy ($\text{h}_{\text{ev}}$) of liquid water and the sensible heat enthalpy ($\text{C×m×ΔT}$, where the specific heat capacity of water $\text{C}$ is 4.2 J g^-1^ K^-1^). The theoretical value of the phase change enthalpy is calculated using equation (S3):

|  | $\text{h}_{\text{e}\text{v}}\text{=}\text{C}_{\text{1}}\text{+}\text{C}_{\text{2}\text{ }}\text{T+}\text{C}_{\text{3}\text{ }}\text{T}^{\text{1}\text{.5}}\text{+}\text{C}_{\text{4}\text{ }}\text{T}^{\text{2}\text{.5}}\text{+}\text{C}_{\text{5}\text{ }}\text{T}^{\text{3}}$ | (S3) |
| --- | --- | --- |

Where $\text{C}_{\text{1}}$, $\text{C}_{\text{2}}$, $\text{C}_{\text{3}\text{ }}$, $\text{C}_{\text{4}\text{ }}$, and $\text{C}_{\text{5}\text{ }}$are 2500.304, -2.2521025, -0.021465847, 3.1750136$\text{×}\text{10}^{\text{-4}}$, and -2.8607959$\text{×}\text{10}^{\text{-}\text{5}}$, respectively, and *T* is the phase change temperature.

The actual phase change enthalpy $\text{h}_{\text{S}}$ of water differs from the theoretical value $\text{h}_{\text{e}\text{v}}$, necessitating determination through equivalent evaporation enthalpy measurements. Thermogravimetric analysis (TGA) is employed to record the constant-temperature mass change rates of pure water and water-containing samples. First, 5 μL of pure water and an equal volume of water-immersed sample are separately dispensed into the ceramic crucible of the TGA instrument, with the sample cut to match the surface area of the pure water. The ceramic crucibles are then transferred to the heating chamber, and the mass change rates at the phase transition temperature are recorded, denoted as $\text{m}_{\text{ev}}$ and $\text{m}_{\text{s}}$, respectively. Assuming that water evaporation is driven by the same energy input $\text{U}_{\text{in}}$, $\text{h}_{\text{S}}$, can be determined using equation (S4):

|  | $\text{U}_{\text{i}\text{n}}\text{=}\text{h}_{\text{e}\text{v}}\text{×}\text{m}_{\text{e}\text{v}}\text{=}\text{h}_{\text{s}}\text{×}\text{m}_{\text{s}}$ | (S4) |
| --- | --- | --- |

**Test S2 Photocatalytic performance under multivariable conditions**

For simulated dye wastewater degradation, RhB was selected as the target pollutant at an initial concentration of 10 ppm. A 50 mL aliquot of RhB solution was placed in a beaker, followed by immersion of hydrogels (5 mg mL⁻¹).

To investigate the salinity effect on photocatalytic performance, solar irradiance was maintained at 1 kW m⁻², with NaCl concentrations of 0 wt% and 10 wt% incorporated into the RhB solution for comparative testing of CB@NFC/SA and PCN-224/CB@NFC/SA hydrogels.

To evaluate temperature and solar irradiance effects, experiments were conducted at controlled temperatures (30 °C, 40 °C, 50 °C) and solar irradiance levels (1.0 kW m⁻²). Additionally, after optimizing the feeding ratio, experiments were carried out under irradiance levels of 0.5, 1.0, and 1.5 kW m⁻² to examine the influence of solar irradiance on photocatalytic efficiency, using a RhB solution containing 10 wt% NaCl at a controlled temperature of 50 °C. All trials were preceded by a 30-minute dark adsorption phase to eliminate interference from adsorption processes.

Samples were collected at 10-minute intervals, centrifuged, and the residual dye concentration in the supernatant was quantified by UV-Vis spectrophotometry.

The degradation percentage *P* of the RhB dye is defined by the formula (S5):

|  | $\text{P=}\frac{\text{C}_{\text{0}}\text{-}\text{C}_{\text{t}}}{\text{C}_{\text{0}}}$ | (S5) |
| --- | --- | --- |

Where *C_0_* is the initial concentration and *C_t_* is the instant concentration of the RhB dye after irradiation. Notably, the impact of water evaporation on the concentration calculation is negligible. Based on the evaporation rate of 2.04 kg m^-2^ h^-1^ and the evaporator surface area (4 cm^-2^), the total water loss during the 60-minute photocatalytic test is approximately 0.816 mL. This represents only ~1.02% of the initial volume (80 mL), a marginal change that falls within the experimental error margin and does not significantly interfere with the degradation efficiency measurements.

**Test S3 Characterization**

Rheological behaviors in the inks were tested by a rheometer (Modular Compact Rheometer:302e, Anton Paar, Austria). The morphologies and microstructures of samples were investigated by scanning electron microscope (SEM) (SU8010, HITACHI, Japan), and an energy-dispersive spectrometer (EDS) was applied to the same instrument. The chemical composition of the samples was analyzed by Fourier transform infrared (FTIR) (Nicolet 877, Thermo Fisher Scientific Co., Ltd., US). The X-ray diffraction (XRD) (Bruker D8 ADVANCE, BRUKER, Germany) data were collected using the Rigaku D/max diffraction system with Cu-Kα radiation (λ = 0.15406 nm). The UV-vis spectra of the samples were measured on a UV-vis spectrophotometer (UV3600, Shimadzu Corporation, Japan). The infrared camera (FLIR T6xx series, FLIR Systems, Inc., US) captured thermal images of the solar-heated samples. The PL spectra of PCN-224 were recorded on the powder PCN-224 by using Fluorescence Lifetime Spectrometers (QM/TM, Power Integrations, US). The Brauer-Emmett-Teller (BET) surface area of PCN-224 was determined with a High-Speed Automated Surface Area and Pore Size Analyzer (Autosorb-iQ, Quantachrome Instruments, US) using N^2^ adsorption isotherms at 120 ℃. The size distribution and zeta potential of PCN-224 were obtained by using the Nano Particle Size Potentiometer (Zetasizer Nano ZS, Malvern Panalytical, UK). Thermal stability was characterized using a TGA apparatus (TGA2-8600, METTLER TOLEDO, Switzerland) under a nitrogen atmosphere. The water contact angle of the samples was measured by a basic dynamic and static contact angle meter (SL200KS, KINO Scientific Instrument Inc., US). Thermal degradation behaviors of the PCN-224 and the printed sample were studied by using the UV–visible diffuse reflectance spectra of the RhB dye was measured using a UV–visible spectrophotometer (UV-1800 spectrometer, Shimadzu Co.Ltd, Japan). The content of the metal element was obtained by inductively coupled plasma optical emission spectrometry (iCAP Q, Thermo Fisher Scientific Co.Ltd, US).

**Supplementary Figures and Tables**

**
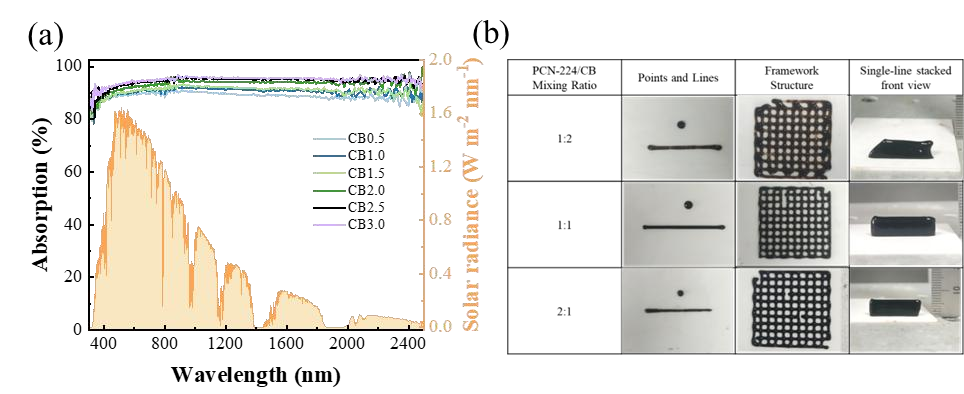
**

**Fig. S1** **a** The effect of different CB loading amounts on the light absorption rate of the composite gels. **b** Visual assessment of the ink printability at different PCN-224 to CB mixing ratios


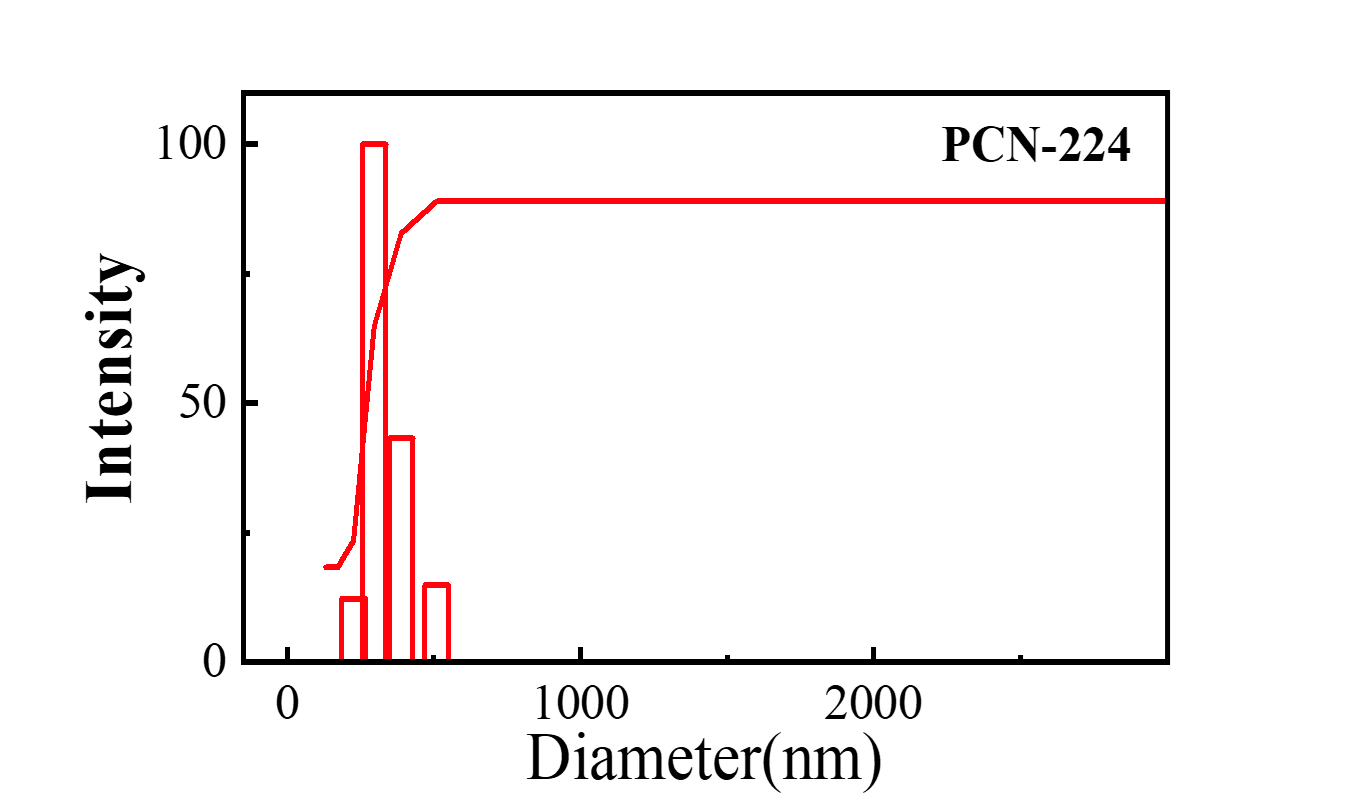


**Fig. S2** Particle size distribution of PCN-224

**
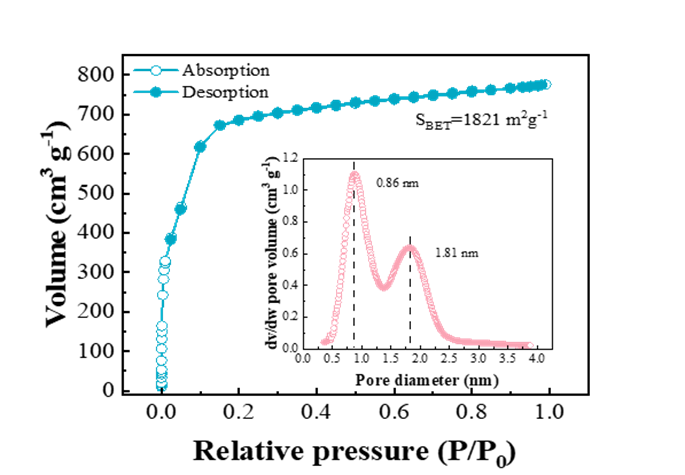
**

**Fig. S3** N_2_ adsorption isotherms at 77 K and pore size distribution of PCN-224 obtained under N_2_ adsorption conditions

**
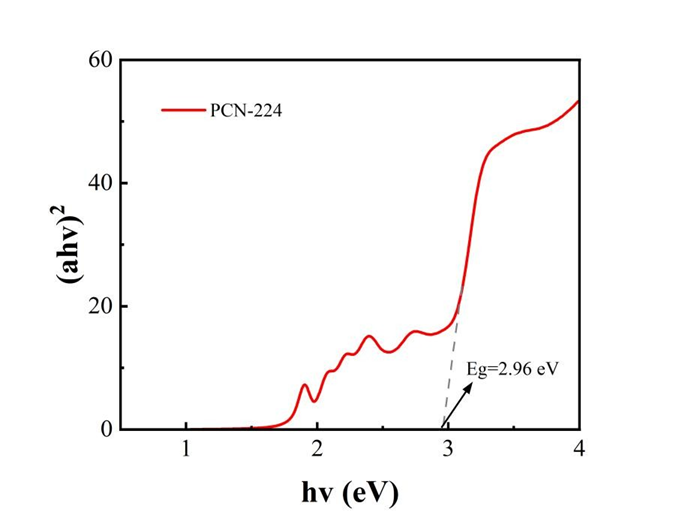
**

**Fig. S4** Energy band gap diagram of PCN-224

**
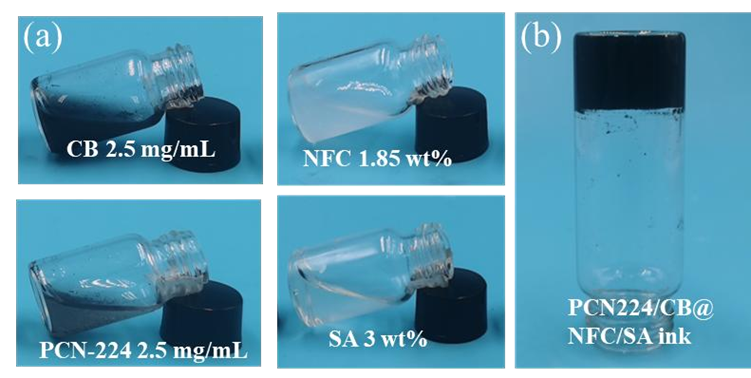
**

**Fig. S5 a** Pictures of CB, PCN-224, NFC, and SA slurry flow. **b** Picture of PCN-224/CB@NFC/SA ink slurry flow

**
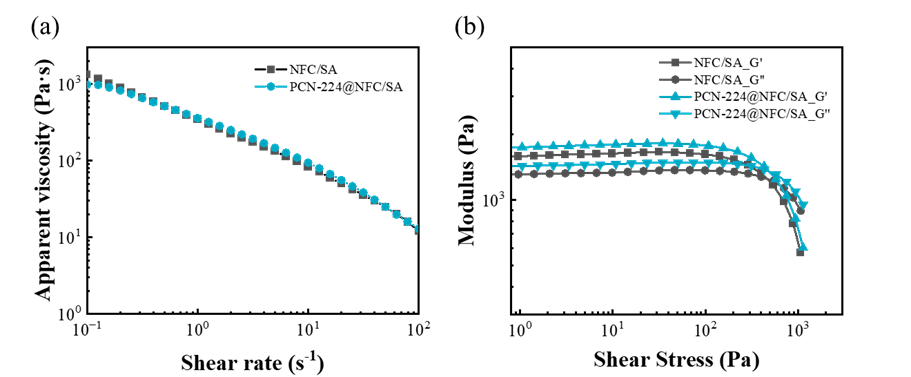
**

**Fig. S6 a** Shear-thinning behavior of two inks. **b** Storage modulus (G′) and loss modulus (G″) of the two inks as a function of shear stress

**
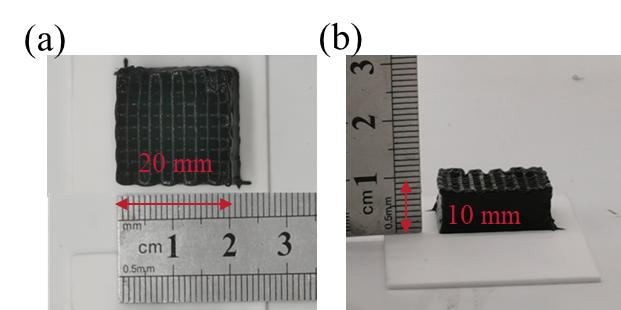
**

**Fig. S7** Pictures of the shape and size of the frame structure: **a** top view. **b** side view

**
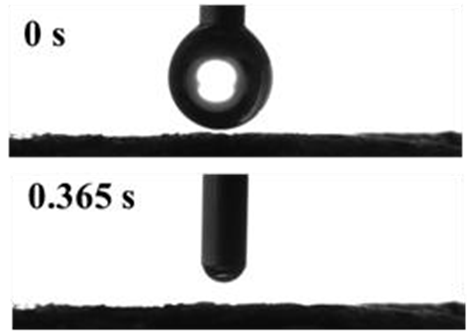
**

**Fig. S8** The water contact angle of PCN-224/CB@NFC/SA

**Table S1** Volume expansion ratio of the PCN-224/CB@NFC/SA composite after equilibrium swelling in different media

| Testing Medium | Volume expansion ratio (Mean±SD, %) |
| --- | --- |
| Pure Water | 24 ± 1.82 |
| 10% NaCl Brine | 5 ± 1.16 |


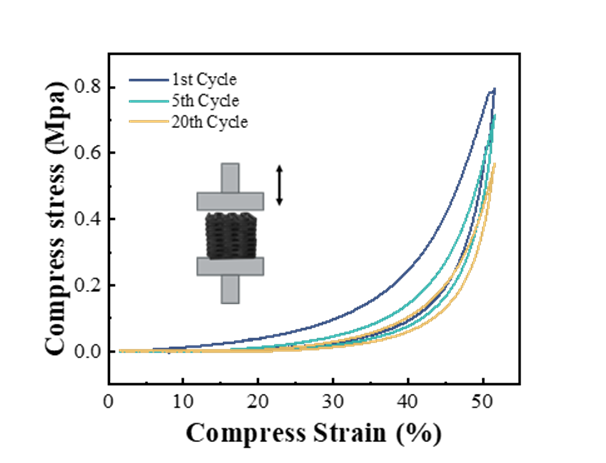


**Fig. S9** Compression loop performance of PCN-224/CB@NFC/SA

**Test S4 The Synergistic Effects of CB and PCN-224**

To investigate whether there is a synergistic effect between PCN-224 and CB, the optical properties of the two composite gels, PCN-224@NFC/SA and PCN-224/CB@NFC/SA, were compared. UV-Vis diffuse reflectance spectroscopy (UV-Vis DRS) testing revealed that the introduction of CB significantly enhanced the absorption capacity of the PCN-224/CB@NFC/SA composite material across the entire visible light to near-infrared regions (Fig. S10a). Additionally, optical bandgap calculations using the Tauc-Plot method revealed that the optical bandgap value (Eg = 0.92 eV) of PCN-224/CB@NFC/SA was significantly reduced compared to PCN-224@NFC/SA (Eg = 1.76 eV) (Fig. S10b).

These phenomena indicate that the interaction between PCN-224 and CB is not merely a physical mixture but involves substantial chemical interactions. The enhanced absorption can be attributed to the synergistic effect of CB's excellent broadband light-harvesting capability and PCN-224. The reduction in optical bandgap typically indicates changes in the electronic structure of the material, which may be due to electronic interactions at the interface between the two materials, leading to band renormalization. More importantly, the significant reduction in the fluorescence intensity of the PCN-224/CB composite material (Fig. S10c) directly demonstrates the efficient transfer of photo-generated electrons from PCN-224 to CB, effectively suppressing the recombination of electron-hole pairs.


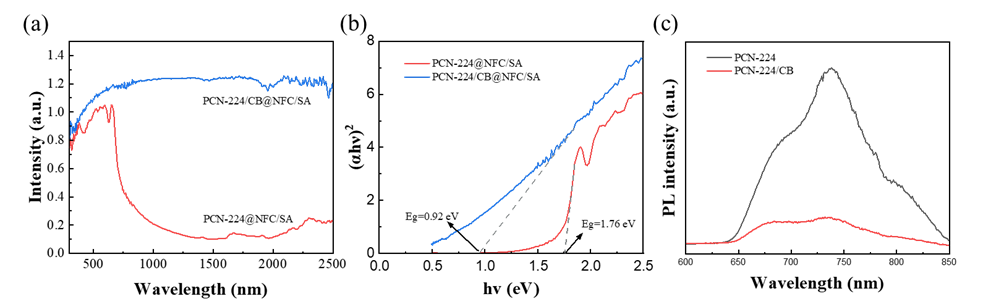


**Fig. S10** UV-vis absorption spectra **a** and Tauc-Plot curves **b** of PCN-224@NFC/SA and PCN-224/CB@NFC/SA. **c** PL spectra of PCN-224 and PCN-224/CB


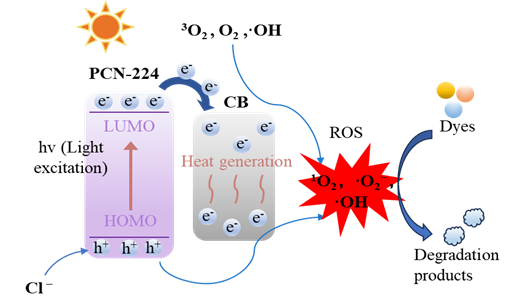


**Fig. S11** Proposed energy band structure and charge transfer mechanism of the PCN-224/CB composite


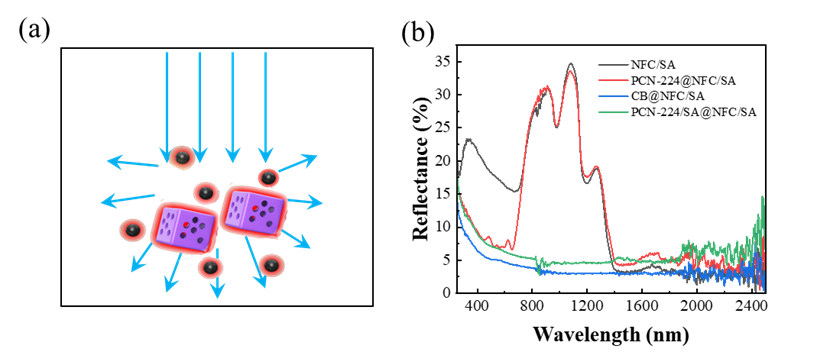


**Fig. S12 a** Schematic representation of light reflection by carbon black and PCN-224 particles. **b** Reflectance of the four hydrogels


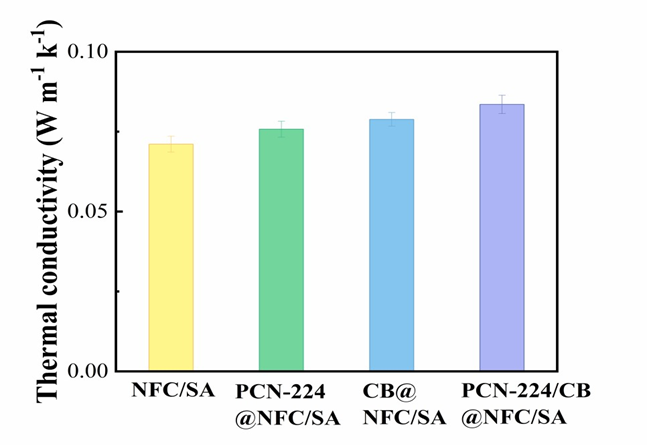


**Fig. S13** Thermal conductivity of four hydrogels


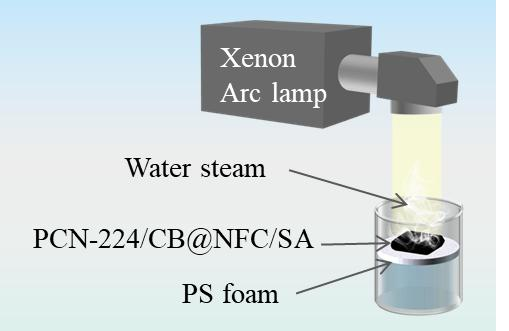


**Fig. S14** Schematic diagram of the device for photo-thermal water evaporation from the hydrogel

**
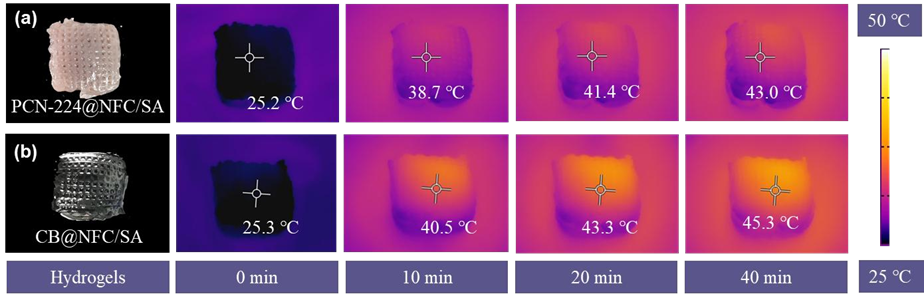
**

**Fig. S15** Infrared images of **a** PCN-224@NFC/SA and **b** CB@NFC/SA under one solar illumination for 40 min

**Test S5 Evaluation of Line Spacing on Evaporation Performance**

**Printing Parameters and Structural Design**

To ensure the fluidity of the printing slurry and the mechanical support of the formed structure, a nozzle with an inner diameter of 0.55 mm was selected, and the printing pressure was maintained constant at 30 psi. This process enabled the stabilization of the printed fiber diameter at *D* ≈ 930 μm. Three distinct initial center-to-center distances (1 mm, 2 mm, and 3 mm) were designed for printing the lattice structures (Fig. S16a).

**Evaporation Performance and Drying-Induced Shrinkage**

Post-testing evaluation revealed that samples with an initial spacing of 2 mm exhibited the optimal evaporation performance (Fig. S16b). It is important to note that significant shrinkage occurred during the drying and curing process. Consequently, the actual average spacing, denoted as “*z*”, was measured to be approximately 1 mm for these samples. All subsequent analyses are based on the actual measured dimensions unless otherwise stated.

**Theoretical Framework for Vapor Diffusion Analysis**

While internal pores (nanoscale to microscale) in porous evaporators contribute to thermal insulation and capillary wicking, they also introduce substantial resistance to vapor diffusion due to their size being significantly smaller than the vapor boundary layer thickness. The calculation of the boundary layer thickness (*δ*_b_) in this study adheres to the theoretical model developed by Zhao et al. [S3].

This model establishes a quantitative relationship between *δ_b_*, fiber diameter (*D*), and fiber spacing (*z*) for AFP-derived aerogel grids. The core formula, derived from Shape Factor theory, is given by:

|  | $\text{δ}_{\text{b}}\text{=}\frac{\left( \text{z+D} \right)\text{·ln(}\frac{\text{8(}\text{z+D}\text{/2)}}{\text{πD}}\text{)}}{\text{2π}}$ | (S6) |
| --- | --- | --- |

A schematic illustrating the relationships between δb, z, and D is provided in Fig. S16c. Furthermore, based on Equation (6), a geometric design window (0.28 < *z*/*D* < 4.77) was defined to ensure low vapor diffusion resistance, originating from the physical constraint *z* > 2*δ*_b_.

**Analysis of Optimal Spacing**

For the sample with an initial 2 mm spacing (actual *z* ≈ 1000 μm, *D* ≈ 930 μm), calculation via Equation (6) yields *δ*_b_ ≈ 427 μm. The critical condition for unimpeded vapor diffusion, *z* > 2*δ*_b_, is satisfied [1000 μm > 2*(427 μm) ≈ 854 μm], confirming efficient vapor escape and minimal diffusion resistance.

Initial 1 mm spacing: The actual spacing z is likely less than 2*δ*_b_, causing boundary layer overlap and consequently higher vapor diffusion resistance.

Initial 3 mm spacing: While the condition *z* > 2*δ*_b_ may be met, an excessively large *z*/*D* ratio reduces the density of evaporative active fibers per unit area, ultimately decreasing the total available evaporation surface and limiting performance.


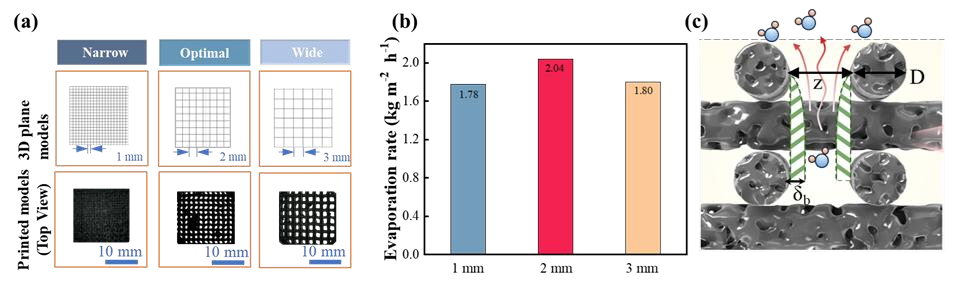


**Fig. S16** **a** 3D printed models and physical photos with different line spacing (1 mm, 2 mm, 3 mm). **b** Evaporation rates for different line spacings (1 mm, 2 mm, 3 mm). **c** Schematic diagram of boundary layer thickness (*δ*_b_), line spacing (*z*), and line diameter (*D*)


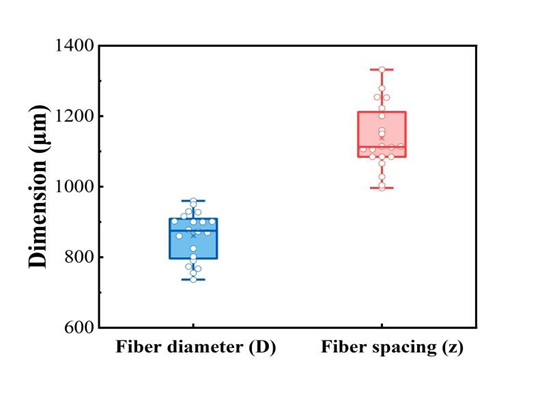


**Fig. S17** Statistical distribution of the geometric dimensions of the 3D-printed lattice


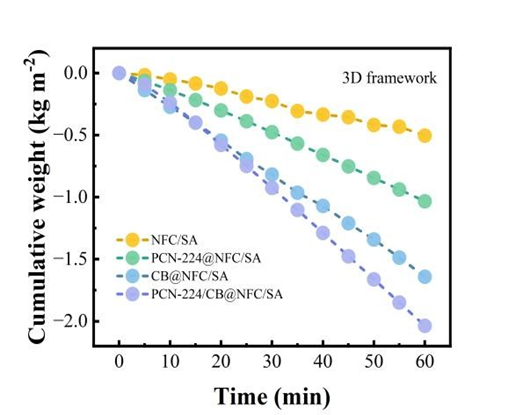


**Fig. S18** Mass change of NFC/SA, PCN-224@NFC/SA, CB@NFC/SA, and PCN-224/CB@NFC/SA evaporated over time


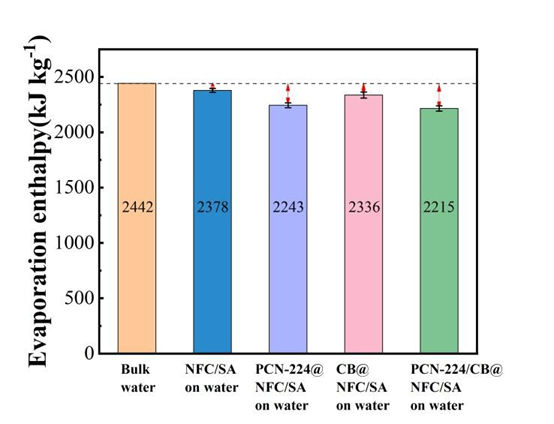


**Fig. S19** Enthalpy of evaporation of five materials


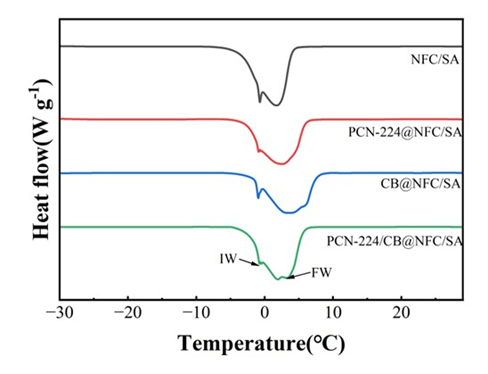


**Fig. S20** DSC melting curves of frozen water in various hydrogel samples

**Test S6 Assessment of Structural Integrity and Mechanical Durability**

**Structural Stability under Wet-Dry Cycling**

The 3D-printed lattice structure's integrity is crucial for sustained water/salt transport. Wet-dry cycling tests (Fig. S21) confirmed that the lattice structure remained intact without any cracking or collapse, ensuring the long-term stability of water transport channels.


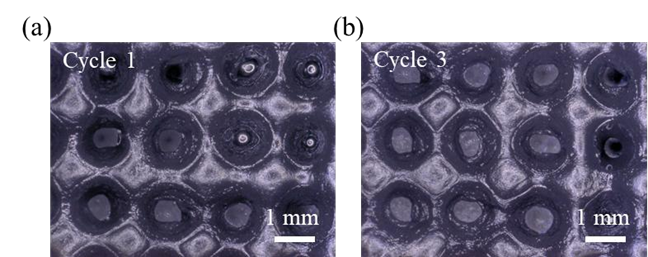


**Fig. S21** Structural evolution of the PCN-224/CB@NFC/SA hydrogel under wet-dry cycling. High-depth-of-field microscopy images after **a** the 1st cycle and **b** the 3rd cycle

**Mechanical Durability under Prolonged Illumination**

To simulate solar-induced aging, the samples were subjected to 12 hours of continuous Xenon lamp irradiation. After irradiation, the hydrogel maintained a high strength retention rate of 87.9% after 20 cycles in the wet state (Fig. S22). Notably, the wet-state retention rate exceeded that of the dry state (71.4%). This is attributed to the plasticizing effect of water molecules: they reduce stress concentration and enhance the flexibility of NFC/SA chains, thereby preventing brittle fracture of the framework under cyclic loading.


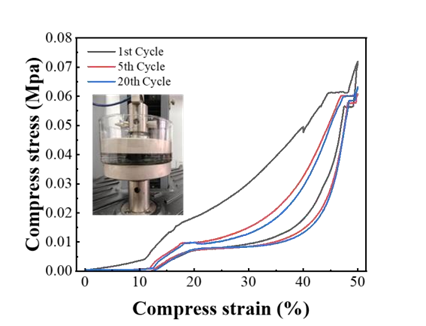


**Fig. S22** Cyclic stress-strain curves (50% strain, 20 cycles) of PCN-224/CB@NFC/SA hydrogel in an aqueous environment after continuous Xenon lamp irradiation for 12 h

**Test S7 Quantitative Analysis of Structural and Ion Transport Properties**

**Porosity and Apparent Density**

The porosity of the porous monoliths is:

|  | $\varepsilon=\left( 1-\frac{\varepsilon_{0}}{\varepsilon_{s}} \right)\times100\%$ | (S7) |
| --- | --- | --- |

where *ε* is the porosity, *ε*_0_ is the apparent density of the porous monoliths, and *ε*_s_ is the volume density of the skeleton of porous monoliths [S4, S5]. The apparent density (*ε*_0_) of the porous monoliths was calculated by measuring the ratio of dry weight to the volume of the samples. The skeletal density of cellulose and SA was assumed to be approximately 1.5 and 1 g cm^-3^, respectively [S6, S7]. Calculations show that the porosity of the 3D-printed framework and solid block is 95.4% and 93.53%, respectively.

**Ion Transport Resistance and Tortuosity**

As shown in the newly added EIS analysis (Fig. S23), the ion transport resistance (*R*_ct_) of the 3D framework (~ 300 Ω) is significantly lower than that of the solid block control (which exhibits a near-vertical slope with resistance exceeding 5000 Ω).


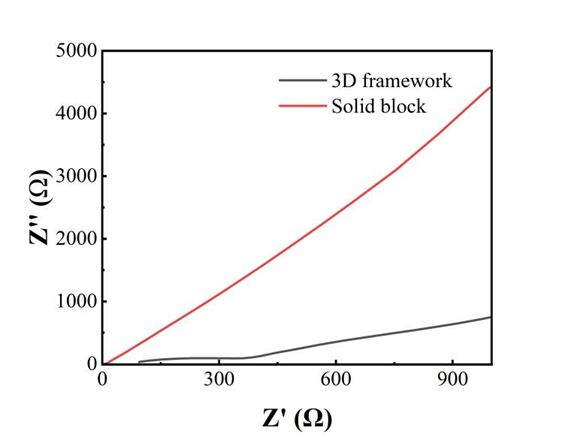


**Fig. S23** Comparative Nyquist plots of the 3D-printed framework and the solid block hydrogel

To further quantify the structural efficiency, the tortuosity (*τ*) was calculated using the MacMullin relationship:

|  | $\tau=\sqrt{\frac{R_{ion}\cdot\varepsilon\cdot\sigma\cdot A}{L}}$ | (S8) |
| --- | --- | --- |

Where *R*_ion_ is the resistance, σ is the electrolyte conductivity, *ε* is the porosity, A is the cross-sectional area, and *L* is the sample thickness. Despite having similar total porosities, the calculated tortuosity of the solid block is approximately 4.2 times higher than that of the 3D framework. This quantifies that the disordered pores in the solid block create a much longer and more obstructed diffusion path for salt ions.

**Summary of Quantitative Parameters**

The following table summarizes the key physical and electrochemical parameters, providing a clear comparison between the two structures:

**Table S2** Quantitative comparison of structural and transport properties

| Parameters | 3D Printed Framework | Solid Block Control | Ratio (3D vs Solid) |
| --- | --- | --- | --- |
| Dimensions (L × W × H) | 20 × 20 × 10 mm | 20 × 20 × 10mm | - |
| Porosity (*ε*, %) | 95.40% | 93.53% | ≈ 1.02 |
| Ion Resistance (*R*_ct_, Ω) | ≈ 300 | > 5000 | > 16.7 |
| Estimated Tortuosity (*τ*) | ≈ 1.25 | ≈ 6.29 | ≈ 5 |
| Salt Transport Flux (kg m^-2^ h^-1^) | 4.125 | 0.75 | 5.5 |


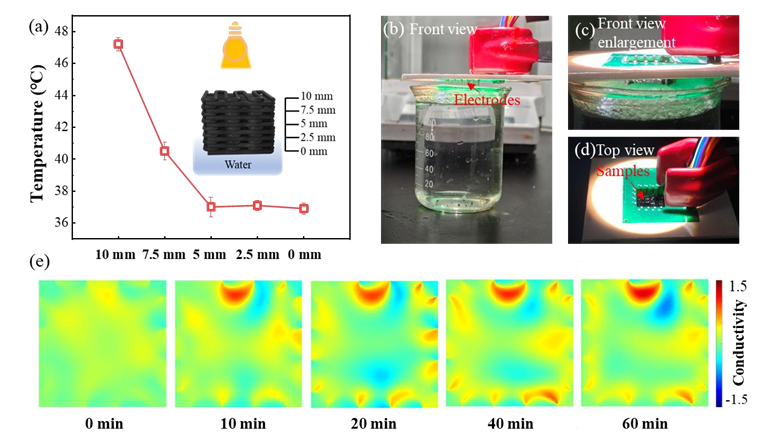


**Fig. S24** Experimental validation of the thermo-solutal Marangoni effect for active salt rejection. **a** Temperature variations at different heights of the evaporator. **b** Front-view of the Electrical Impedance Tomography (EIT) testing setup. **c** Enlarged front-view of the interface. **d** Top-view of the testing setup. **e** EIT conductivity distribution map at 60 min

**Test S8 Experimental Validation of Salt Transport Flux**

To quantitatively validate the enhanced salt back-flow in our 3D-printed framework, the salt transport flux experiments comparing it to a non-porous solid block were conducted (Fig. S25), following the method reported by Yang et al. [S8].


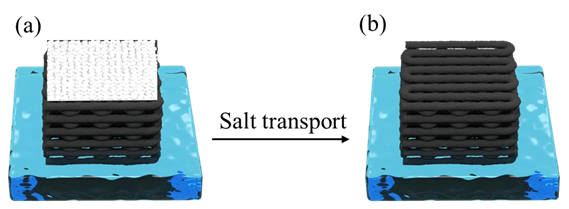


**Fig. S25** Schematic diagrams of the salt flux measurement process. **a** The state of the initial evaporator. **b** The state of the final evaporator

Briefly, 0.5 g NaCl was evenly spread on the surface of the dry evaporator, which was then brought into contact with brine at an initial concentration *C_1_* (3.5 wt%) to start the timing. Within a period of time *T* (10 min), the salt within the porous structure of the evaporator diffused into the bulk brine driven by the concentration gradient. The salt flux *J* was calculated based on the change in mass *m (*25 g*)* and concentration *C_2_* of the bulk brine before and after the experiment using the following formula (S9):

|  | $\text{J=}\frac{\text{m(}\text{C}_{\text{2}}\text{-}\text{C}_{\text{1}}\text{)}}{\text{S}\text{·T}}$ | (S9) |
| --- | --- | --- |

Where *S* is the effective mass transfer area of the evaporator, and *T* is the measurement time.

Under identical test conditions (m = 25 g, C₁ = 3.5%, S = 4 cm², T = 10 min), calculations show that the salt flux of the 3D-printed porous framework (4.125 kg m⁻² h⁻¹) is significantly higher than that of the solid block (0.75 kg m⁻² h⁻¹). This result directly demonstrates that the 3D-printed porous framework structure is more conducive to salt reflux. Its vertical grid structure provides a large specific surface area and abundant capillary channels, greatly enhancing the transport efficiency of salts from the evaporation interface to the bulk solution through capillary convection and diffusion. This is the fundamental reason for its outstanding salt transport capability.

**The Critical Role of Low-Tortuosity Pores: A Literature Corroboration**

The decisive role of pore geometry is further strongly supported by independent work. Yang et al [S9]. constructed a 3D evaporator with vertically aligned Mass Transfer Bridges (MTBs)—a design paradigm of low tortuosity. Their device achieved stable operation in high-salinity brines (12–14 wt% NaCl) without salt accumulation, sustaining a high evaporation rate (~1.64 kg m⁻² h⁻¹). This work directly confirms that low-tortuosity channels are the key to enabling efficient salt back-flow, which aligns perfectly with our findings.

Regarding the Dedye length, we performed calculations based on the experimental conditions described in the paper (saline concentration of 3.5 wt%, approximately 0.61 M NaCl). The Debye length (*λ_D_*) of NaCl is calculated using the formula (S10):

|  | $\text{λ}_{\text{D}}\text{=}\sqrt{\frac{\text{ε}_{\text{r}}\text{ε}_{\text{0}}\text{k}_{\text{B}}\text{T}}{\text{2}\text{e}^{\text{2}}\text{c}_{\text{0}}}}$ | (S10) |
| --- | --- | --- |

Where: *ε_r_* is the relative permittivity of water (~80), *ε_0_* is the permittivity of free space, *k_B_* is the Boltzmann constant, T is the absolute temperature (typically 298 K at room temperature), *e* is the elementary charge, and *c₀* is the volume concentration of the electrolyte (m⁻³).

Calculations indicate that λ_D_ is approximately 0.39 nm in this system. The pore size distribution of our material (hydrogel), as determined by SEM characterization data (Fig. 2i in the paper), ranges from 20,000 to 80,000 nm. Clearly, even using the most conservative estimate (minimum pore size of 20 μm), 20,000 nm >> 0.39 nm.

This substantial size ratio (d_pore_ >> λ_D_) implies that the electrostatic double layer effect within the pore is confined to the sub-nanometer region immediately adjacent to the pore walls, while the vast majority of the pore space constitutes an electrically neutral bulk solution region [S10]. Consequently, ion transport within the pore is unaffected by nanoconfinement effects or ion selectivity, and its reflux kinetics are primarily governed by the diffusion coefficient of the bulk solution and the geometric structure of the pore (such as tortuosity).

**Table S3** Dynamic salt transport flux of the 3D framework under hypersaline conditions and fluctuating salt loads

| Time Interval (min) | Operational Status | Bulk Salinity (wt%) | Salt Flux (J, kg m^-2^ h^-1^) |
| --- | --- | --- | --- |
| 0-10 | 0.5 g NaCl added | 10.0-10.6 | 2.25 |
| 10-20 | - | 10.6-10.6 | 0 |
| 20-30 | 0.5 g NaCl added | 10.6-11.4 | 3.00 |
| 30-40 | - | 11.4-11.6 | 0.75 |
| 40-50 | 0.5 g NaCl added | 11.6-12.4 | 3.00 |
| 50-60 | - | 12.4-12.6 | 0.38 |

**Test S9 Adsorption Performance of PCN-224 for RhB**

PCN-224 is a porous metal-organic framework material. To determine its adsorption equilibrium time for RhB, which will guide subsequent photocatalytic experimental conditions, adsorption kinetics tests were conducted in this study. Under dark conditions at room temperature, PCN-224 was interacted with a 200 ppm RhB solution, with a contact time set to 120 minutes. The changes in the adsorption amount of RhB by PCN-224 at different time points were monitored to clarify the adsorption equilibrium time and investigate its adsorption kinetic behavior.

Based on the experimental measurements, the kinetic profile of RhB adsorption was clearly revealed. The results showed (Fig. S26a) that the adsorption of RhB by PCN-224 reached equilibrium within approximately 10 minutes. To ensure that the adsorption process reached complete equilibrium before the photocatalytic experiments, a uniform pre-adsorption time of 30 minutes was adopted in subsequent experiments to minimize the influence of adsorption effects on the photocatalytic degradation process.

To gain further insight into the adsorption mechanism, the adsorption process was fitted using pseudo-first-order and pseudo-second-order kinetic models, with the model equations shown below:

|  | Pseudo-first-order kinetic model: | $\text{Q}_{\text{t}}\text{=}\text{Q}_{\text{e}}\text{×(1}\text{- }\text{e}^{\text{-}\text{K}_{\text{1}}\text{t}}\text{)}$ | (S11) |
| --- | --- | --- | --- |
|  | Pseudo-second-order kinetic model: | $\frac{\text{t}}{\text{Q}_{\text{t}}}\text{=}\frac{\text{1}}{\text{k}_{\text{2}}\text{Q}_{\text{e}}^{\text{2}}}\text{+}\frac{\text{t}}{\text{Q}_{\text{e}}}$ | (S12) |

In the formula, *Q_e_* and *Q_t_* represent the adsorption amounts of simulated pollutants at equilibrium and adsorption time *t* (min), respectively; *K_1_* (min^-1^) is the adsorption rate constant of the pseudo-first-order model, and *K_2_* (g (mg min)^-1^) is the kinetic constant of the pseudo-second-order model.

The fitting parameters are listed in Table S4. The pseudo-second-order kinetic model exhibited a higher correlation coefficient (R²), indicating that the adsorption behavior of PCN-224 toward RhB better conforms to the pseudo-second-order kinetic model, with the adsorption mechanism primarily dominated by chemical adsorption.


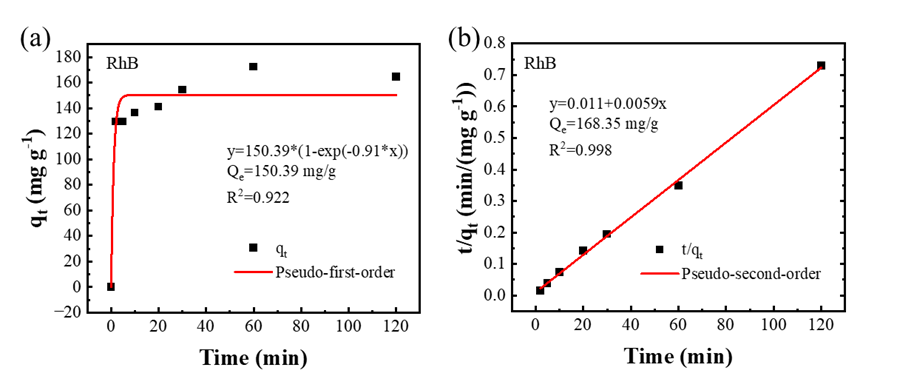


**Fig. S26** **a** Pseudo-first-order kinetic model fitting results of RhB. **b** Pseudo-second-order kinetic of RhB

**Table S4** Adsorption kinetic model fitting parameters of PCN-224 toward RhB.

| Sample | Pollutant | Pseudo-first-order | | | Pseudo-second-order | | |
| --- | --- | --- | --- | --- | --- | --- | --- |
|  |  | Q_1e_  (mg/g) | K_1_  (/min) | R^2^ | Q_2e_  (mg/g) | K_2_  (g/mg/min) | R^2^ |
| PCN-224 | RhB | 150.39 | 0.91 | 0.922 | 168.35 | 0.0033 | 0.997 |

**Photocatalytic Performance of PCN-224 for RhB**

To verify the photocatalytic performance of PCN-224 toward RhB, 50 mg of PCN-224 was dispersed in 100 mL of RhB solution with an initial concentration of 200 ppm, and stirred in the dark for 30 minutes to achieve adsorption equilibrium. Subsequently, photocatalytic reactions were conducted under 500 W xenon lamp irradiation. Samples were collected every 30 minutes, centrifuged, and the UV-Vis absorption spectra of the supernatant in the 400–700 nm range were measured.

The results showed that the intensity of the characteristic RhB absorption peak at 554 nm gradually decreased with increasing irradiation time (Fig. S27a); blank experiments indicated that RhB was almost not degraded in the absence of a catalyst (Fig. S27b)); simultaneously, the solution containing PCN-224 exhibited a significantly lighter color (Fig. S27c), confirming that PCN-224 exhibits a significant photocatalytic degradation effect on RhB.

**
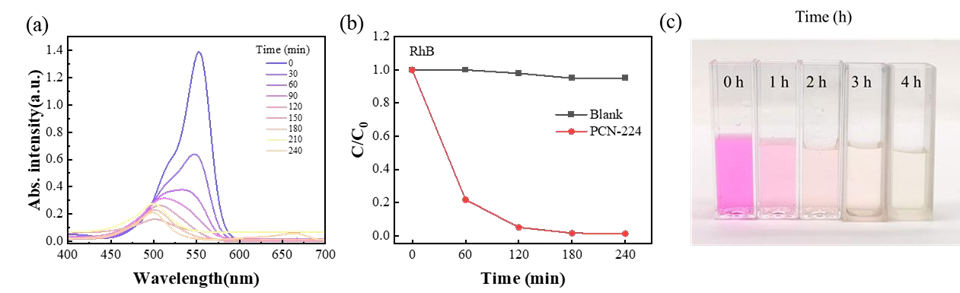
**

**Fig. S27 a** UV-Vis spectra of the RhB solution with PCN-224 under light irradiation. **b** Photodegradation efficiency of RhB with and without PCN-224. **c** Photograph showing the corresponding color changes of the solution over time


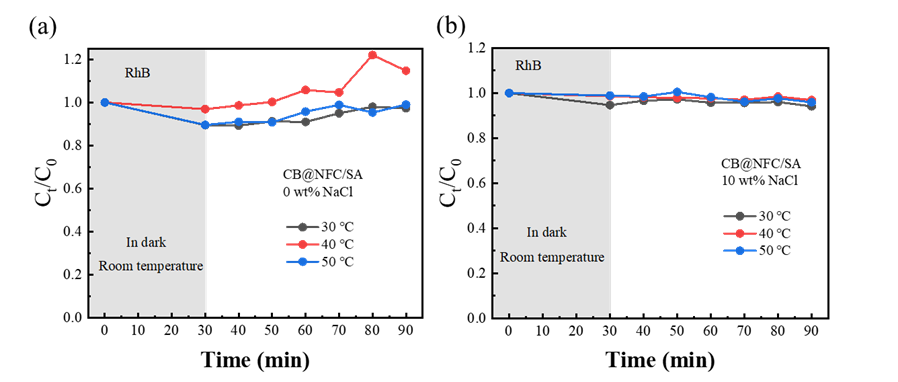


**Fig. S28** The degradation rates of RhB in **a** 0 wt% NaCl, **b** 10 wt% NaCl by CB@NFC/SA

**Test S10 Chemical Stability and Structural Fidelity in High-Salinity Environments**

**Quantitative Analysis of Metal Ion Leaching**

ICP-MS analysis shows that after 24 h of immersion in a 10% NaCl solution at a loading of 0.1 mg mL^-1^, the concentration of leached Zr^4+^ is 0.43 mg L^-1^. This translates to a mass leaching ratio of approximately 1.43%, whereas the Zr concentration in pure water remained below the detection limit. This marginal leaching rate in an environment with nearly three times the salinity of typical seawater demonstrates the robust nature of the Zr-O clusters within the PCN-224 framework.

**Qualitative Evaluation of Crystalline Integrity**

Furthermore, the PXRD patterns (Fig. S29) of the samples recovered from the brine show sharp diffraction peaks that perfectly match the pristine material. There is no evidence of phase transformation or crystallinity loss.


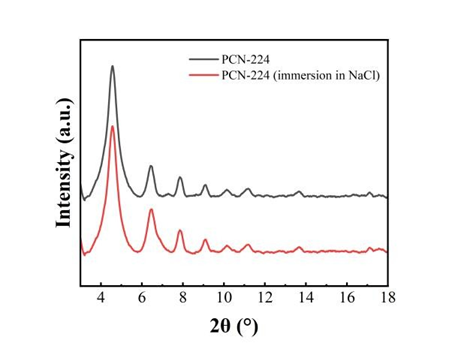


**Fig. S29** PXRD patterns of PCN-224 before and after 24 h of immersion in 10% NaCl solution


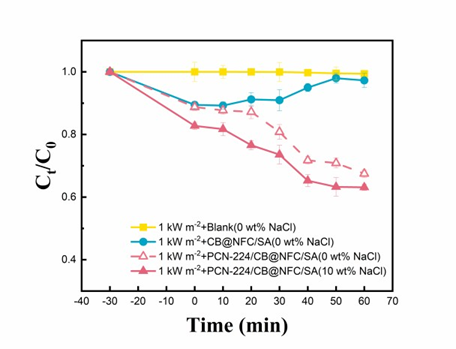


**Fig. S30** Degradation of RhB under different conditions

**Test S11 Catalytic Cycle Stability of PCN-224**

The cyclic stability of PCN-224 for RhB degradation was evaluated over three consecutive cycles. The initial concentration of RhB is 10 ppm (10 wt% NaCl) and the PCN-224 dosage ratio is 0.1 mg mL⁻¹. After each cycle (30 min adsorption in the dark followed by 60 min under 1.5 kW m^-2^ light irradiation), the catalyst was recovered by centrifugation, thoroughly washed with ethanol to remove any residual organic species, and dried before the next run.

As Fig. S31a, the degradation efficiency for RhB remained at 84.58% in the first cycle, 82.33% in the second cycle, and 79.87% in the third cycle. The slight decrease in activity can be attributed to the inevitable minor mass loss during the recovery and washing process rather than catalyst deactivation. This result confirms the high operational stability and reusability of our PCN-224 catalyst.

To further corroborate the structural stability, we characterized the fresh and used catalyst (after 3 cycles) by XRD and FT-IR. As shown in Fig. S31b, the XRD pattern of the used PCN-224 catalyst after three cycles is nearly identical to that of the fresh one. All characteristic diffraction peaks of PCN-224 are well-preserved, indicating that the crystalline framework and structural integrity of PCN-224 were maintained throughout the photocatalytic process.

As shown in Fig. S31c, the FT-IR spectra of the fresh and used catalysts also show no significant changes. First, the positions of key characteristic peaks (~1584 cm⁻¹ and ~1404 cm⁻¹) attributed to Zr₆ clusters and carboxylate coordination bonds remained stable, indicating that the core framework of the PCN-224 remained intact after the catalytic reaction. Second, the vibration peaks attributed to Zr-O bonds (~650 cm⁻¹ and ~474 cm⁻¹) were clearly visible, with no new characteristic peaks associated with organic contaminants appearing. These results conclusively demonstrate that PCN-224 exhibits outstanding structural and chemical stability during photocatalytic cycling.


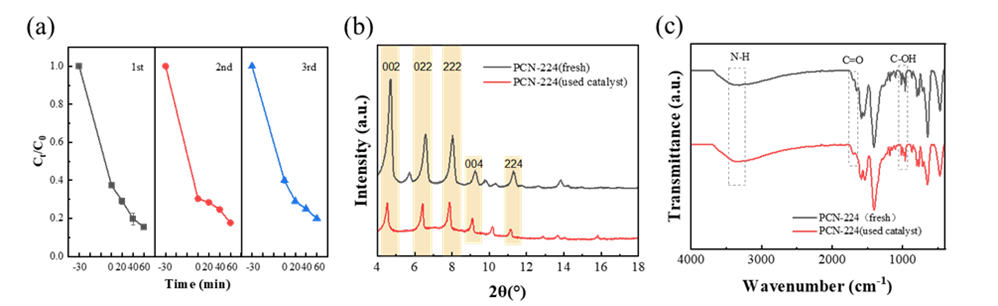


**Fig. S31 a** Cyclic stability of PCN-224 for the photocatalytic degradation of RhB. **b** XRD patterns and **c** FTIR spectra of PCN-224 before and after three consecutive photocatalytic. cycles

**Test S12 Catalytic Cycle Stability of PCN-224/CB@NFC/SA**

The cyclic stability of PCN-224/CB@NFC/SA for RhB degradation was evaluated over three consecutive cycles. After each cycle (30 min adsorption in the dark followed by 60 min under light irradiation), the catalyst was recovered by centrifugation, thoroughly washed with low-concentration ethanol solution to remove any residual organic species, and dried before the next run (Fig. S32a).

During 10 consecutive photocatalytic tests, the degradation efficiency of RhB only decreased slightly from 96.5% to 89%, indicating the robust stability of the PCN-224 photocatalyst within the matrix. The slight decrease in activity can be attributed to the inevitable minor mass loss during the recovery and washing process rather than catalyst deactivation. This result confirms the high operational stability and reusability of our PCN-224/CB@NFC/SA catalyst.

To further corroborate the structural stability, we characterized the fresh and used catalyst PCN-224/CB@NFC/SA (after 3 cycles) by FT-IR. As shown in Fig. S32b, the FT-IR spectra of the fresh and used catalysts PCN-224/CB@NFC/SA also show no significant changes. In the spectra of fresh and used PCN-224/CB@NFC/SA catalysts, the trans-COO- and cis-COO- peaks shifted slightly from 1598 and 1410 cm⁻¹ to 1595 and 1422 cm⁻¹, respectively. This minor shift likely indicates a reversible change in the local coordination environment induced by the catalytic reaction and washing process, but the integrity of the framework remains intact. The absence of new peaks confirms that no major decomposition occurred, underscoring the robust stability of the material.


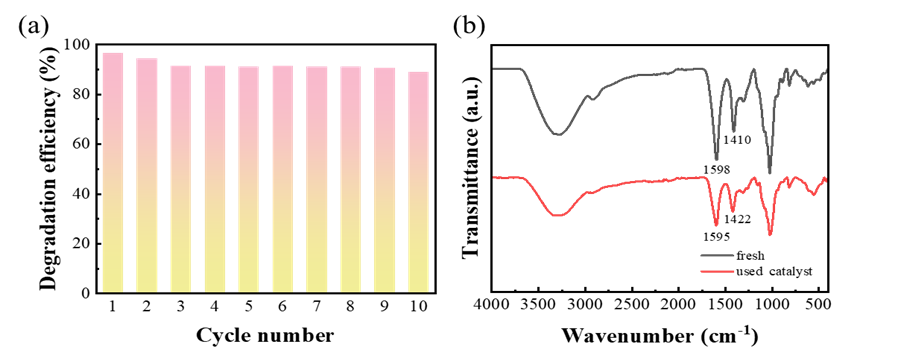


**Fig. S32** **a** Cyclic stability of PCN-224/CB@NFC/SA for the photocatalytic degradation of RhB. **b** FTIR spectra of PCN-224/CB@NFC/SA before and after three consecutive photocatalytic cycles


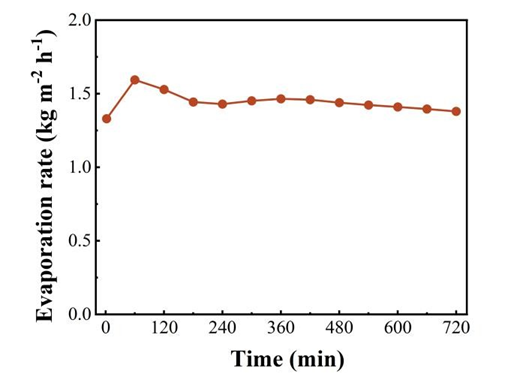


**Fig. S33** Evaporation rate and stability of the PCN-224/CB@NFC/SA hydrogel during 12 h of continuous operation in a 10% NaCl solution containing RhB


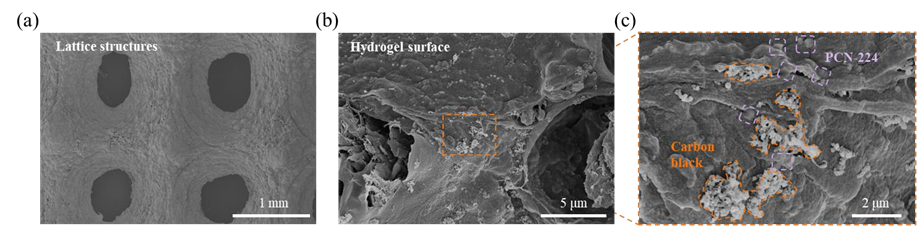


**Fig. S34** Structural characterization of the hydrogel after long-term testing. **a** SEM image of the freeze-dried 3D lattice framework hydrogel after 24 h of operation. **b** SEM image of the hydrogel surface. **c** High-magnification SEM images revealing the stable encapsulation of PCN-224 and CB particles within the NFC/SA polymer matrix


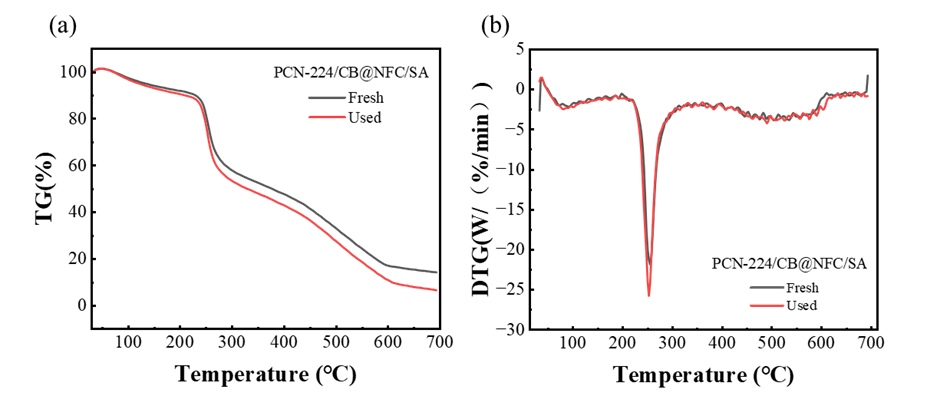


**Fig. S35** **a** TG and **b** DTG curves of the fresh and used PCN-224/CB@NFC/SA hydrogel

**Test S13 Photocatalytic performance of PCN-224/CB@NFC/SA toward Methylene blue (MB)**

Under one-sun irradiation, the photocatalytic efficiency of PCN-224/CB@NFC/SA hydrogel toward MB was evaluated for 60 minutes. As shown in Fig. 36a, in a 0 wt% NaCl solution, the degradation efficiencies of MB at 30, 40, and 50 °C were 84.8%, 88.6%, and 93.0%, respectively. Notably, unlike the enhancing effect observed with RhB, the addition of 10 wt% NaCl significantly inhibited MB degradation, with the degradation efficiency remaining around 73.2% regardless of temperature variation (Fig. S36b).


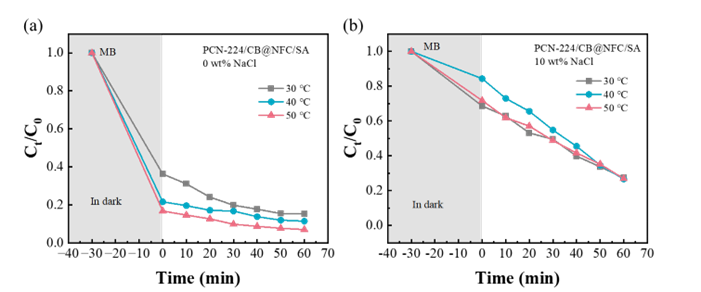


**Fig. S36** Effect of water temperature on the degradation rate of MB catalyzed by PCN-224/CB@NFC/SA under one-sun irradiation: **a** in 0 wt% NaCl solution. **b** in 10 wt% NaCl solution


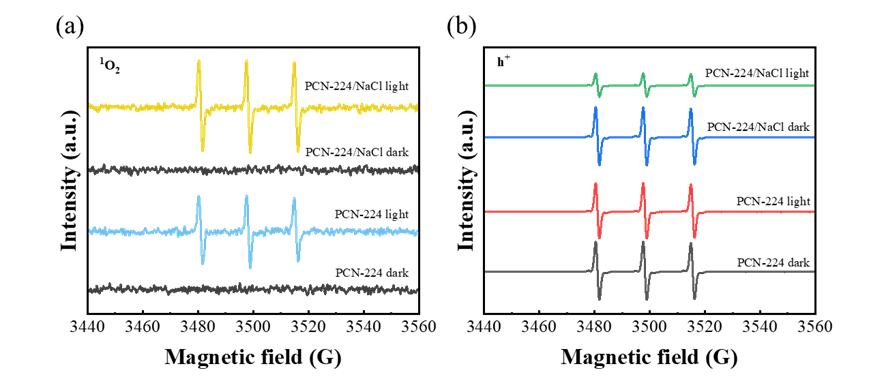


**Fig. S37** EPR spectra of reactive species over PCN-224 under saline (10 wt% NaCl) and salt-free conditions after light irradiation for 10 min: **a** DMPO-·OH in aqueous solution. **b** DMPO-·O^2−^ in anhydrous methanol

**Test S14 Zeta Potential Testing**

The zeta potentials of PCN-224, RhB, and their mixture (PCN-224/RhB) were measured in NaCl solutions at different concentrations (0 wt%, 3.5 wt%, and 10 wt%) (Fig. S32).

The zeta potential of PCN-224 sharply increased from -33.97 mV (0 wt% NaCl) to -6.26 mV (10 wt% NaCl), with an absolute value decrease of 82%. The potential of RhB also increased from -12.91 mV to -4.92 mV, representing a 62% decrease in absolute value. This indicates that high ionic strength significantly compresses the double layer.

Under 0 wt% NaCl conditions, the absolute value of the potential for the PCN-224/RhB mixture (-4.87 mV) was lower than both the potential for PCN-22 alone (-33.97 mV) and the potential for RhB alone (-12.91 mV). This directly demonstrates the successful adsorption of RhB molecules onto the PCN-224 surface.

Salt-Enhanced Adsorption Behavior: At 10 wt% salt concentration, the mixture potential (-20.63 mV) exhibits a larger absolute value compared to the salt-free mixture (-4.87 mV). This indicates that reduced electrostatic repulsion allows for the adsorption of a larger, denser layer of RhB molecules, thereby more fully exposing the dye's negative charge.


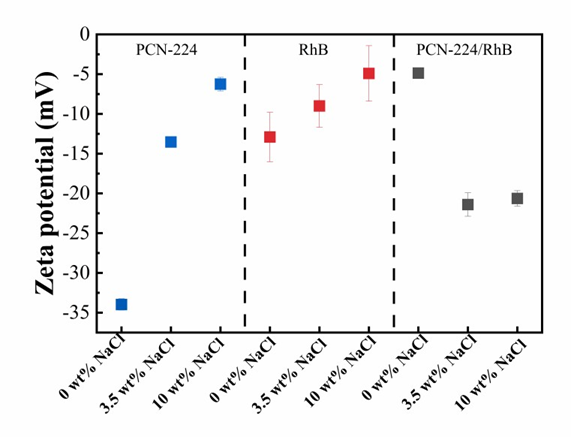


**Fig. S38** Zeta potentials of PCN-224, RhB, and their mixture (PCN-224/RhB) as a function of NaCl concentration

**Test S15 DFT Calculation Details and Methods**

Density functional theory (DFT) calculations were performed using the ORCA program package to investigate the role of Cl⁻ in promoting the adsorption and photocatalytic degradation of Rhodamine B (RhB) on PCN-224. A cluster model was extracted from the experimental crystal structure of PCN-224, consisting of a Zr₆O₈ node coordinated with truncated carboxylate ligands derived from the TCPP linker, in order to reduce the computational cost while preserving the local coordination environment. Two models were constructed:

Model A (Control): Zr₆O₈ cluster with truncated organic ligands + RhB molecule.

Model B (Saline): Zr₆O₈ cluster with truncated organic ligands and an adsorbed Cl⁻ at the Zr site + RhB molecule.

All geometries were optimized using the B97-3c composite method, which includes Grimme’s D3 dispersion correction to properly account for van der Waals interactions. Subsequently, single-point energy calculations were performed using the ωB97M-V functional with the def2-TZVP basis set for all atoms to obtain more accurate electronic energies. Frequency calculations were conducted to confirm that the optimized structures correspond to true minima on the potential energy surface.

The adsorption energy (E_ads_) was calculated according to the following equation (S13):

|  | $E_{\text{ads}}=E_{\text{total}}-(E_{\text{MOF}}+E_{\text{RhB}})$ | (S13) |
| --- | --- | --- |

where $E_{\text{total}}$ is the energy of the MOF-RhB complex, and $E_{\text{MOF}}$ and $E_{\text{RhB}}$ are the energies of the isolated MOF cluster and RhB molecule, respectively.

Charge density difference (CDD) maps were generated using Multiwfn 3.8 and visualized with VMD 1.9.3 to illustrate electron redistribution upon RhB adsorption with and without Cl⁻. AIM charge analysis was performed to quantitatively evaluate charge transfer between RhB and the MOF surface.


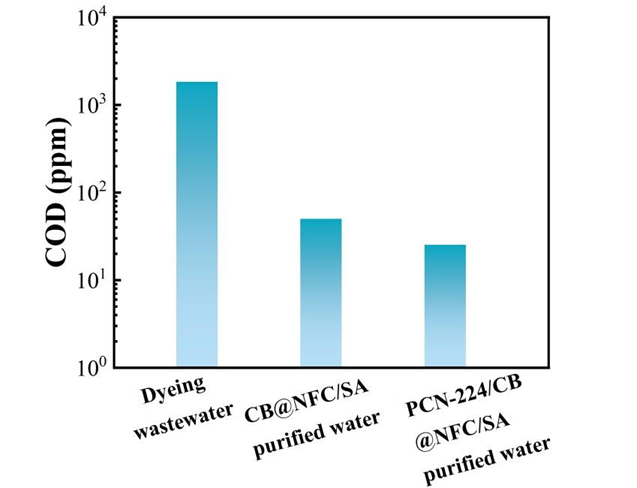


**Fig. S39** Chemical oxygen demand (COD) of real dyeing wastewater before and after purification using the CB@NFC/SA and PCN-224/CB@NFC/SA evaporators


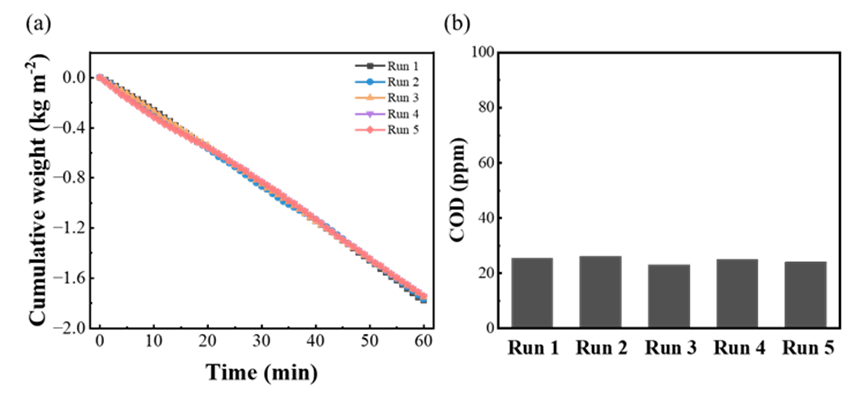


**Fig. S40** Long-term stability and anti-deactivation performance of the PCN-224/CB@NFC/SA evaporator in actual dyeing wastewater. **a** Cyclic evaporation rates over 5 consecutive cycles. **b** COD concentration of the collected condensate across 5 cycles


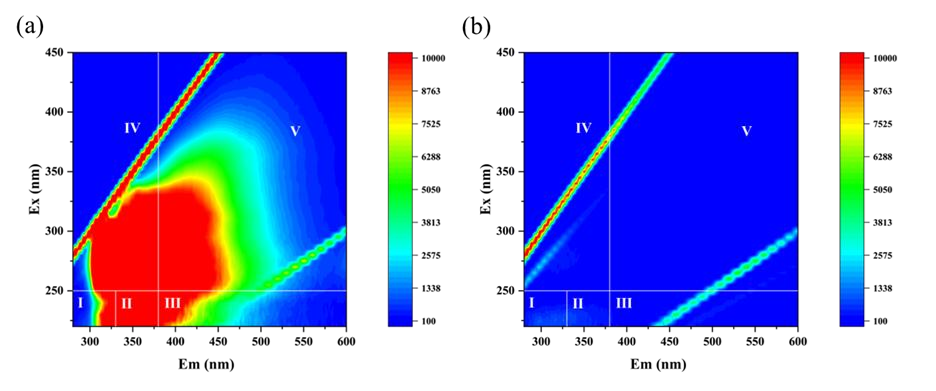


**Fig. S41** 3D-EEM image of **a** the original dyeing wastewater and **b** the purified water

**Table S5** Concentration of heavy metal elements in raw dyeing wastewater and purified water

| Element | Dyeing wastewater（Mean ± SD，mg L^-1^） | Purified water  (Mean ± SD，mg L^-1^) |
| --- | --- | --- |
| Sb | 8.09 ± 0.02 | 0.01 ± 0.01 |
| Zn | 0.08 ± 0.01 | 0.02 ± 0.01 |
| Cr | 0.02 ± 0.01 | 0.02 ± 0.01 |
| Pd | 0.07 ± 0.01 | 0.01 ± 0.01 |
| Cu | 0.03 ± 0.01 | 0.01 ± 0.01 |
| Ni | 0.06 ± 0.01 | 0.01 ± 0.01 |


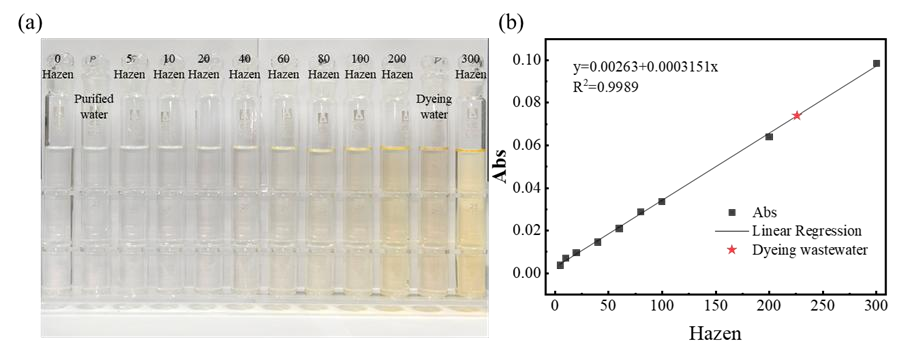


**Fig. S42** Chromaticity analysis via the Pt-Co colorimetric method. **a** Digital photograph showing the color gradient of Pt-Co standard solutions (0–300 Hazen) alongside the dyeing wastewater and purified water. **b** Linear calibration curve of absorbance as a function of colority at 455 nm

**Table S6** PCN-224/CB@NFC/SA hydrogel cost analysis

| Component | Unit price ($ g^-1^) | Mass per unit (g) | Cost per unit ($) | Cost per m^2^ ($) |
| --- | --- | --- | --- | --- |
| PCN-224 | 3 | 0.25 | 0.75 | 75 |
| CB | 0.0002 | 0.25 | 0.00005 | 0.005 |
| SA | 0.01 | 3.0 | 0.03 | 0.75 |
| NFC | 0.1 | 1.0 | 0.10 | 0.25 |
| 3D printing costs | - | - | 0.035 | 3.50 |
| Total |  |  | $ 0.915 | $ 91.5 |

Note: (1) One unit corresponds to an evaporator area of 0.01 m².

(2) The unit prices are estimated based on laboratory-scale bulk synthesis and commercial reagent prices.

**Table S7** Comparison of material cost, fabrication method, and performance with recently reported solar

| Reference | Material | Fabrication method | | Material cost ($ m^-2^) | Evaporation Rate (kg m^-2^ h^-1^) | Photocatalytic |
| --- | --- | --- | --- | --- | --- | --- |
| *Water Res. 2026, 292, 125375* | Hydrothermal carbonization wood (HCPW-230) | Hydrothermal carbonization and freeze-drying | 11.84 | | 2.64 | No |
| *Adv. Mater. 2023, 35, 2304699* | Ni NPs-CNF | 3D printing | ~504 | | 2.08 | No |
| *Adv. Sci. 2024, 11, 2308665* | AC/Starch hydrogel | 3D printing | 10.14 | | 2.13 | No |
| **This work** | **PCN-224/CB@NFC/SA** | **3D printing** | **91.5** | | **2.04** | **Yes** |

**Table S8** Comparison of various evaporators

| Literature | Material system | Manufacturing method | Evaporation rate (kg m^-2^ h^-1^) | Degradation (Target/Time/%) | Salt resistance (Conc./ Stability) |
| --- | --- | --- | --- | --- | --- |
| *Chem. Eng. J., 2022, 430, 132765* | Cellulose/alginate/carbon black hydrogel | 3D printing | 1.33 | MB complete removal (rate not specified) | 3.5 wt% NaCl/Stable |
| *Mater. Today Phys., 2024, 43, 101394* | CNTs-modified photocurable polymer resin | 3D-printed S-shaped channels | 1.40 | Metal ions (Na⁺, K⁺, Mg²⁺, Cu²⁺, Fe³⁺, Zn²⁺): >99.9% removal;  Organic dyes (RhB, methyl blue, citron yellow), residual < 23 mg/L (rate not specified) | 3.5 wt% NaCl/ Stable |
| *J. Clean. Prod., 2026, 545, 147767* | PETMP/PEGDA/MWCNTs-COOH porous polymer | DLP 3D printing | 1.704 | Dye wastewater (reactive red): complete removal (rate not specified) | 2.5-10 wt% NaCl/Stable |
| *Mater. Horiz., 2024, 11(22), 5740-5751* | BiOBr@ZCW | ZIF-67 multi-step hydrothermal synthesis | 1.67 | RhB / 20 min / 98.43% | 25% NaCl & saturated salt / Stable |
| *Adv. Funct. Mater., 2025, 15344* | Ni-PT-COF@wood | Schiff-base reaction | 2.07 | Uranium (U) / 12 h / > 93% | Not reported |
| *Chem. Eng. J., 2025, 505, 159130* | Cu-ZIF-67/rGO aerogel | Thermal reduction and surface growth | 1.95 | MB / 1 h / 78.52% | Simulated seawater/Stable |
| **This work** | **PCN-224/CB@NFC/SA** | **3D-printed lattice** | **2.04** | **RhB/60 min/96.5%;**  **COD (textile wastewater): 1828 → 25 mg L^-1^ / 4 h;**  **Sb (heavy metal): >99% removal** | **3.5%-20% NaCl/Stable** |

**Supplementary References**

1. D. Van-Duong, V. Ngoc Hung, H.-S. Choi, All day limnobium laevigatum inspired nanogenerator self-driven via water evaporation. J. Power Sources **448**, 227288 (2020). [https://doi.org/10.1016/j.jpowsour.2019.227388](%20https:/doi.org/10.1016/j.jpowsour.2019.227388.)
2. V.D. Dao, An Experimental exploration of generating electricity from nature-inspired hierarchical evaporator: the role of electrode materials. Sci. Total Environ. **759**, 143490 (2021**)**. [https://doi.org/10.1016/j.scitotenv.2020.143490](https://doi.org/10.1016/j.scitotenv.2020.143490.)
3. X. Zhao, Y. Yang, X. Yin, Z. Luo, K.-Y. Chan et al., Size-insensitive vapor diffusion enabled by additive freeze-printed aerogels for scalable desalination. Acs Energy Lett. **10**, 3419-3429 (2025**)**. [https://doi.org/10.1021/acsenergylett.5c01233](https://doi.org/10.1021/acsenergylett.5c01233.)
4. T. Zhu, Z. Ren, D. Wang, S. Zhao, X. Liu et al., Reactive 3D printed silanized cellulose nanofiber aerogels for solar-thermal regulatory cooling, Compos. Part A-Appl. S. **192**，108761 (2025). <https://doi.org/10.1016/j.compositesa.2025.108761>
5. C. Liu, X. Shi, B. Lin, Z. Song, F. jiang et al., 3D-printing of hierarchical porous monoliths using emulsion gels stabilized by lignin-containing cellulose nanofibrils for thermal insulation, Carbohyd. Polym. **375**，124787 (2026). <https://doi.org/10.1016/j.carbpol.2025.124787>
6. L. Yang, H. Zhang, C. Wang, Y. Jiao, X. Pang et al., Novel aerogels based on supramolecular G-quadruplex assembly with intrinsic flame retardancy and thermal insulation, J. Colloid Interface Sci. **672**，618-630 (2024). <https://doi.org/10.1016/j.jcis.2024.06.048>
7. G. Zhou, M.-C. Li, F. Wang, C. Liu, C. Mei, 3D printing of cellulose nanofiber monoliths for thermal insulation and energy storage applications, Addit. Manuf. **59**，103124 (2022). <https://doi.org/10.1016/j.addma.2022.103124>
8. H. Yang, Y. Sun, M. Peng, M. Cai, B. Zhao et al., Tailoring the salt transport flux of solar evaporators for a highly effective salt-resistant desalination with high productivity. ACS Nano **16**, 2511-2520 (2022**)**. <https://doi.org/10.1021/acsnano.1c09124.>
9. K. Yang, T. Pan, S. Dang, Q. Gan, Y. Han, Three-dimensional open architecture enabling salt-rejection solar evaporators with boosted water production efficiency. Nat. Commun. **13**, 6653 (2022). [https://doi.org/10.1038/s41467-022-34528-7](https://doi.org/10.1038/s41467-022-34528-7.)
10. A.M. Smith, A.A. Lee, S. Perkin, The electrostatic screening length in concentrated electrolytes increases with concentration. J. Phys. Chem. Lett. **7**, 2157-2163 (2016). [https://doi.org/10.1021/acs.jpclett.6b00867](https://doi.org/10.1021/acs.jpclett.6b00867.)
